# Verteporfin ameliorates fibrotic aspects of Dupuytren’s disease nodular fibroblasts irrespective the activation state of the cells

**DOI:** 10.1038/s41598-022-18116-9

**Published:** 2022-08-17

**Authors:** Nataly Puerta Cavanzo, Sophie A. Riesmeijer, Iris L. Holt-Kedde, Paul M. N. Werker, Bram Piersma, Peter Olinga, Ruud A. Bank

**Affiliations:** 1 Department of Pharmaceutical Technology and Biopharmacy, Groningen Research Institute of Pharmacy, University of Groningen, Antonius Deusinglaan 1, 9713 AV Groningen, The Netherlands; 2 MATRIX Research Group, Department of Pathology and Medical Biology, University Medical Center Groningen, University of Groningen, Hanzeplein 1, 9713 GZ Groningen, The Netherlands; 3 Department of Plastic Surgery, University Medical Center Groningen, University of Groningen, Hanzeplein 1, 9713 GZ Groningen, The Netherlands

**Keywords:** Phenotypic screening, Connective tissue diseases

## Abstract

Dupuytren’s disease is a chronic, progressive fibroproliferative condition of the hand fascia which results in digital contraction. So far, treatments do not directly interfere with the (myo)fibroblasts that are responsible for the formation of the collagen-rich cords and its contraction. Here we investigated whether verteporfin (VP) is able to inhibit the activation and subsequent differentiation of DD nodular fibroblasts into myofibroblasts. Fibroblasts were isolated from nodules of 7 Dupuytren patients. Cells are treated (1) for 48 h with 5 ng/ml transforming growth factor β1 (TGF-β1) followed by 48 h with/without 250 nM VP in the absence of TGF-β1, or treated (2) for 48 h with TGF-β1 followed by 48 h with/without VP in the presence of TGF-β1. mRNA levels were measured by means of Real-Time PCR, and proteins were visualized by means of Western blotting and/or immunofluorescence. Quantitative data were statistically analyzed with GraphPad Prism using the paired t-test. We found that fibroblasts activated for 48 h with TGF-β1 show a decrease in mRNA levels of *COL1A1*, *COL3A1*, *COL4A1*, *PLOD2*, *FN1EDA*, *CCN2* and *SERPINE1* when exposed for another 48 h with VP, whereas no decrease is seen for *ACTA2*, *YAP1, SMAD2* and *SMAD3* mRNA levels. Cells exposed for an additional 48 h with TGF-β1, but now in the presence of VP, are not further activated anymore, whereas in the absence of VP the cells continue to differentiate into myofibroblasts. Collagen type I, fibronectin-extra domain A, α-smooth muscle actin, YAP1, Smad2 and Smad3 protein levels were attenuated by both VP treatments. We conclude that VP has strong anti-fibrotic properties: it is able to halt the differentiation of fibroblasts into myofibroblasts, and is also able to reverse the activation status of fibroblasts. The decreased protein levels of YAP1, Smad2 and Smad3 in the presence of VP explain in part the strong anti-fibrotic properties of VP. Verteporfin is clinically used as a photosensitizer for photodynamic therapy to eliminate abnormal blood vessels in the eye to attenuate macular degeneration. The antifibrotic properties of VP do not rely on photo-activation, as we used the molecule in its non-photoinduced state.

## Introduction

Dupuytren’s disease (DD) is a fibroproliferative condition of the hand fascia that affects mainly adults of Northern European ascent^1,2^. The etiology of the disease is unclear, but genetic predisposition plays an important role. Studies in twin siblings show heritability up to 80%^3^ and genome-wide association studies show specific genomic regions that give rise to DD^4–6^. Moreover, several non-genetic factors are reported to contribute to the development of the disease, such as smoking, alcohol intake, diabetes, hyperlipidemia and manual labor or exposure to vibrations^1,2,6,7^.

DD is characterized by the presence of fibrotic lesions that seem to spread along the palmar fascia of the palm and fingers, that may contract thereby impairing their extension, thus affecting hand function and quality of life^1,2,6,7^. The disease has three different stages, all of which can be present at the same time. The initial proliferative stage is when nodules form, containing numerous myofibroblasts, driving the whole fibrotic response. The proliferative stage is followed by an involutional stage, where myofibroblasts and extracellular matrix (ECM) are aligned along the stress lines of the hand. It ends with the residual stage, where the nodules disappear, leaving behind a mostly acellular fibrotic tendon-like structure (cord) that adheres to the skin and fascia resulting in contracture lesions^1,2,6^.

Treatment of DD comprises nonsurgical treatments (e.g. physiotherapy, intralesional injected steroids or radiotherapy) which are usually performed in early stages of the disease, but the efficacy of these treatments is not clear^1,8,9^. A novel treatment approach is the injection of collagenase derived from *Clostridium histolyticum*. Its efficacy continues to be evaluated^10–12^. The late stage contractures are treated by surgical interventions such as fasciotomy, fasciectomy or dermofasciectomy and skin grafting^1,2,6,7,13,14^*.* However, all of the above interventions frequently lead to recurrences^15–17^. Unfortunately, as is the case with fibrosis in general, an effective pharmacological treatment has not been developed yet.

Although DD is a tissue-specific fibrosis, it seems likely that it behaves on a cellular and molecular level in a similar way as other chronic fibrotic processes in different organs of the body. These processes are the result of many different biochemical and biophysical cues. These cues are sensed by fibroblasts, which subsequently are triggered to differentiate into myofibroblasts. The latter cells express α-smooth muscle actin (α-SMA), resulting in the formation of stress fibers that increase cellular contractibility. Simultaneously, myofibroblasts show increased synthesis of extracellular matrix (ECM) proteins such as collagen types I and III and fibronectin-extra domain A (Fn-EDA). Under fibrotic conditions, the balance between the production and degradation of extracellular matrix proteins becomes impaired. The balance is, apart from the production of ECM itself, also determined by the interplay between matrix metalloproteinases and tissue inhibitors of metalloproteinases^6,18^.

Several molecular pathways have been associated with the onset of DD. Among them, the transforming growth factor β (TGF-β) pathway is highly relevant, as it is in other fibrotic disorders. The profibrotic cytokine TGF-β is involved in the differentiation of fibroblasts towards myofibroblasts, including the maintenance of the activation status of myofibroblasts through the intracellular downstream Smad signaling cascade. Stimulation of the TGF-β pathway results in the phosphorylation of Smad2 and Smad3, forming a complex that binds to Smad4, a complex that in turn activates the transcription of fibrosis-related genes^19–21^. TGF-β levels are increased in DD fibroblasts compared to control fibroblasts, along with its downstream effectors Smad2 and Smad3. As a consequence, ECM markers are also increased. Stimulation with TGF-β1 in vitro increases myofibroblast proliferation, collagen contraction, as well as synthesis of ECM components^22–24^.

Three important intertwined signal transduction routes, namely the transforming growth factor (TGF)-β, the Wingless/Int (WNT), and the yes-associated protein 1 (YAP)/transcriptional coactivator with PDZ-binding motif (TAZ) pathways, have been linked to the pathophysiology of fibrosis^25^. GWAS studies have shown that several components of the WNT pathway are linked to DD^4–6^, that TGFβ1 is highly expressed in affected DD tissues^22–25^, and more recently that YAP is highly expressed in Dupuytren nodules^26^.

Several preclinical studies revealed that specifically inhibiting the YAP/TAZ pathway by e.g. siRNA results in a major improvement in the outcome of fibrosis^27,28^. Unfortunately, translation of preclinical fibrosis models into a clinical therapy has often failed, e.g., due to major species differences. Apart from that, introduction of a new drug into the clinic is an expensive and time-consuming process. However, it was recently found that verteporfin (VP), a FDA-approved benzoporphyrin derivative that is already used in the clinic to inhibit macular degeneration, is capable of inhibiting extracellular matrix deposition in liver, kidney and retinal rodent fibrosis models by interfering with the YAP/TAZ pathway^18,29–31^.

By using mouse and rat fibroblasts under various (fibrotic) conditions, it was found that VP inhibits the production of collagen (the hallmark of fibrosis) as well as that of many other profibrotic molecules^29,32–35^ . A few studies also investigated the effect of VP on human cells in the context of fibrosis (retinal Müller cells; ventricular fibroblasts; Peyronie plaque fibroblasts; conjunctival fibroblasts; dermal fibroblasts; systemic sclerosis dermal fibroblasts)^31,36–39^. In all cases, a decrease is seen in collagen production, either on mRNA level or protein level. Of these studies, only a single paper investigated the effect of VP in human cells at both the mRNA and protein level^39^. Here we report the effect of VP on nodular fibroblasts from surgically excised tissue of 7 DD patients, both at the mRNA and protein level. Since the aim of our study is to evaluate the anti-fibrotic potential of VP in both the early and late stage of the disease, we created a profibrotic environment by stimulating DD cells with TGF-β1 for 48 h before VP treatment. This was followed by a comparison between DD fibroblasts treated with VP for another 48 h under continued TGF-β1 stimulation (highly activated fibroblasts) with DD fibroblasts without further TGF-β1 stimulation (i.e., fibroblasts with a lower activation level). Our results show a significant decrease of fibrotic markers, both at the mRNA and protein level in both experimental groups, and conclude that VP has major anti-fibrotic properties.

## Methods

### Reagents for cell culture

Reagents and final concentrations used in culture medium were as follows: Human recombinant TGF-β1 (5 ng/ml; 100-21C, Peprotech, London, UK); ascorbic acid (0.17 mM; A8960, Sigma-Aldrich, Merck KGaA, Darmstadt, Germany); penicillin/streptomycin (pen/strep) (50 U/L; 15,140,122, Thermo Fisher Scientific, Landsmeer, The Netherlands), verteporfin (250 nM; SML0534, Sigma-Aldrich, The Netherlands). Stock solutions of the drug VP were prepared in dimethyl sulfoxide (DMSO) and stored at − 20 °C; the working solutions were diluted in culture medium with a final solvent concentration of ≤ 1%.

### Cell culture

**Figure 1:**
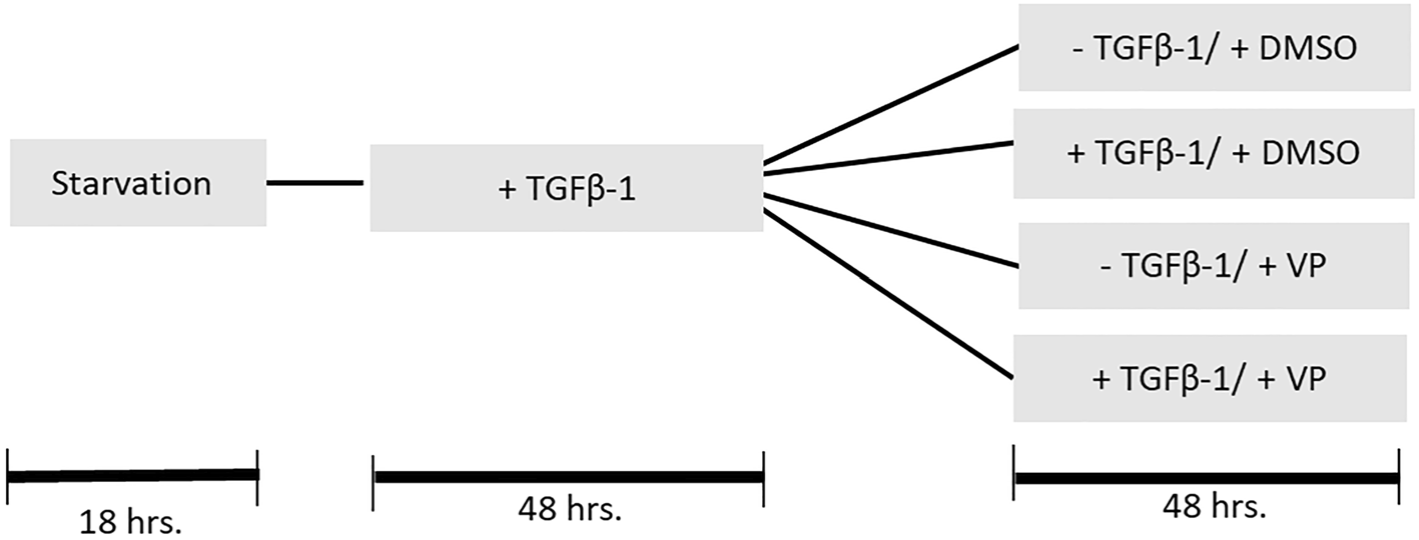
Schematic representation of treatments subjected to DD nodule-derived fibroblasts. DMSO is used as the control, as VP is prepared in DMSO.

Dupuytren tissue was obtained from 7 patients undergoing primary limited fasciectomy or dermofasciectomy (Caucasians from the northern part of the Netherlands; age range 59–67). Informed written consent was obtained through approval of the local ethics committee. Surgical pieces were macroscopically dissected into nodule and cord. Nodules were separated in pieces of 0.5 cm^2^, four pieces per well were placed in 6 well plates. Isolation of primary nodule fibroblast was performed by the outgrow method in Minimum Essential Medium – Eagle with Earle's BSS (MEM Eagle EBSS; 12-611F, Lonza) containing 20% fetal bovine serum (FBS). FBS was filtered before use through a SFCA 0.45 μm filter. Before the onset of experiments, Dupuytren nodular fibroblasts were propagated in Dulbecco’s modified Eagle medium (DMEM) (12-604F, Lonza) containing 50 U/L pen/strep) and 10% FBS (Sigma-Aldrich). All cells were tested negative for mycoplasm. At the start of the experiment, cells were trypsinized, reseeded at a density of 15.000 cells/cm^2^, and starved for 18 h in DMEM containing 50 U/L pen/strep, 0.5% FCS and 0.17 mM ascorbic acid. Experiments were carried out in primary fibroblasts passage 4 to 6 with DMEM containing 50 U/L pen/strep, 0.17 mM ascorbic acid, and 0.5% FBS. Treatments were performed as follows (Fig. 1): for all cells, stimulation with 5 ng/ml TGF-β1 in starvation medium for 48 h. Next, cells were treated for 48 h in starvation medium with or without 5 ng/ml TGF-β1 stimulation in the presence of 1% (v/v) DMSO (= control vehicle) or 250 nM VP. Medium was refreshed every 24 h.

### RNA extraction and Real-Time PCR

For gene expression analysis, total RNA was isolated with the Tissue Total RNA mini kit (Favorgen Biotech Corp., Taiwan). RNA quantity and quality were determined by UV spectrophotometry (NanoDrop Technologies, Wilmington, DE). One μg of RNA was reverse transcribed with RevertAid First Strand cDNA Synthesis kit (Thermo Fischer Scientific). Real-time PCR was performed with SYBR green PCR master mix (Roche, Basel, Switzerland) and VIIA7 thermal cycling system (Applied Biosystems, Carlsbad, CA). Thermal cycling conditions were 2 min at 95 °C (enzyme activation), followed by 15 s at 95 °C, 30 s at 60 °C and 30° seconds at 72 °C (40 cycles). Melting curve analysis was performed in order to verify the absence of primer dimers. Primers were designed and optimized to have a calculated 95% to 105% reaction efficiency; the used sequences are shown in Table 1. All data were normalized against the reference gene tyrosine 3-monooxygenase/tryptophan 5-monooxygenase activation protein, zeta isoform (*YWHAZ*). The following genes were measured: the extracellular matrix proteins collagen type I, III, IV and fibronectin-extra domain A (*COL1A1*, *COL3A1*, *COL4A1* and *FN1EDA*, respectively), the collagen modifying enzyme lysyl hydroxylase 2 (*PLOD2*), the cytoskeletal protein alfa-smooth muscle actin (*ACTA2*), the matricellular protein connective tissue growth factor (*CTGF* = *CCN2*), the serpin family protein plasminogen activator inhibitor-1 (*PAI1* = *SERPINE1*), and three Hippo/TGFβ pathway proteins (*YAP1*, *SMAD2* and *SMAD3*).

**Table 1:** Primer sequences used for q-PCR.

Gene	Forward sequence	Reverse sequence
*COL1A1*	GCCTCAAGGTATTGCTGGAC	ACCTTGTTTGCCAGGTTCAC
*COL3A1*	AGGGTGCAATCGGCAGTCCA	CAATGGCAGCGGCTCCAACA
*COL4A1*	TGGTGACAAAGGACAAGCAG	GGTTCACCCTTTGGACCTG
*FN1EDA*	AATCCAAGCGGAGAGAGTCA	GGAATCGACATCCACATCAG
*PLOD2*	GGGAGTTCATTGCACCAGTT	GAGGACGAAGAGAACGC
*ACTA2*	CTGTTCCAGCCATCCTTCAT	TCATGATGCTGTTGTAGGTGGT
*CCN2*	AGCTGACCTGGAAGAGAACATT	GCTCGGTATGTCTTCATGCTG
*SERPINE1*	TGGTGCTGATCTCATCCTTG	AGAAACCCAGCAGCAGATTC
*YAP1*	AATCCCACTCCCGACAGG	GACTACTCCAGTGGGGGTCA
*SMAD2*	TCACTCCATTCCAGAAAACACTAA	ATATCCAGGAGGTGGCGTTT
*SMAD3*	CACCACGCAGAACGTCAA	GATGGGACACCTGCAACC
*YWHAZ*	GATCCCCAATGCTTCACAAG	TGCTTGTTGTGACTGATCGAC

### Immunofluorescence

Detection of both intracellular and extracellular collagen type I, α-SMA and fn-EDA isoform was carried out on fixed cells with 4% paraformaldehyde (Sigma-Aldrich) for 10 min. Later they were permeabilized with 0.5% Triton X-100 in phosphate-buffered saline (PBS) for 10 min. For all immunostainings, fixed cells were subsequently incubated with PBS + 0.1% Triton, containing 2.2% bovine serum albumin (BSA, Sanquin Reagents, Amsterdam, The Netherlands) and 5% of specific anti-serum antibody for 60 min at room temperature (RT). The primary antibodies were incubated for 120 min at RT. The secondary antibodies were incubated for 30 min at RT. The binding protein Streptavidin Cy3 in PBS + 1% BSA was incubated in the dark for 20 min at RT. Nuclei were visualized with 4′,6-diamidine-2′-phenylindole dihydrochloride (DAPI) (dilution 1:5000). All wash steps were performed in PBS-Tween. The cells were mounted with Citifluor AF1 (AF1-25, Brunschwig Chemie, Amsterdam, The Netherlands). Microphotographs were acquired in a random blind fashion with the use of a Leica TCS SP8 confocal system microscope (Leica Microsystems, Rijswijk, The Netherlands). Antibodies used are shown in Table 2.

**Table 2:** Used antibodies in immunofluorescence (IF) and Western blotting (WB).

Target	Company	Catalog number	Application	Concentration
Mouse anti-human collagen type I	Abcam, Cambridge, United Kingdom	ab90395	IF	1:1000
Mouse anti-human α-smooth muscle actin (α-SMA)	Dako, Glostrup, Denmark	M0851	IF, WB	1:500
Mouse anti-human fn-EDA	Abcam, Cambridge, UK	ab6328	IF	1:1000
Goat anti-human collagen type I	Santa Cruz Biotechnology, Dallas, Texas, USA	sc-8783	WB	1:1000
Mouse anti-human YAP	Santa Cruz Biotechnology, Dallas, Texas, USA	sc-101199	WB	1:500
Mouse anti-human Smad2	Cell Signaling Technology, Leiden, The Netherlands	3103S	WB	1:1000
Rabbit anti-human Smad3	Abcam, Cambridge, UK	Ab28379	WB	1:1000
Rabbit Anti- human YWHAZ	Abcam, Cambridge, UK	ab51129	WB	1:1000
Biotinylated, goat Anti-mouse IgG	SouthernBiotech, Birmingham, AL, USA	1031–08	IF	1:500
Streptavidin-CY3	SouthernBiotech, Birmingham, AL, USA	7100–12	IF	1:1000
Polyclonal Goat anti-rabbit Immunoglobulins/HRP	Dako, Glostrup, Denmark	P0448	WB	1:5000
Polyclonal Rabbit anti-goat Immunoglobulins/HRP	Dako, Glostrup, Denmark	P0449	WB	1:5000
Polyclonal Goat anti-mouse Immunoglobulins/HRP	Dako, Glostrup, Denmark	P0447	WB	1:5000

### Immunoblotting

Cells were lysed with RIPA buffer (Thermo Fischer Scientific) supplemented with 1% protease inhibitor cocktail (Sigma-Aldrich) and 1% phosphatase inhibitor cocktail (Sigma-Aldrich), and lysates were disrupted with sonication. Protein concentrations were determined with a DC protein assay (Bio-Rad, Hercules, CA), and equal amounts of protein (30 µg/ml) per lane were subjected to SDS gel electrophoresis. Protein transfer to a nitrocellulose membrane was performed with the semidry Transblot Turbo system (Bio-Rad). Membranes were blocked in Tris-buffered saline (TBS) + 0.5% Tween20 (Sigma-Aldrich) that contained 5% milk powder. Primary and secondary antibodies were diluted in TBS-Tween20 that contained 5% milk powder. Membranes were cut prior to incubation with primary and secondary antibodies. Primary antibodies were incubated overnight at 4 °C, secondary antibodies were incubated for 60 min at RT. All washing steps were performed in TBS-Tween20. Protein bands were visualized with chemiluminescence and ChemiDoc imaging system (Bio-Rad). Image analyses were performed with Image Lab™ Software version 6.0.1 (Bio-Rad). Original images of blots from all the replicates are included in the supplementary information, acquisition was performed when the total length of the ladder was visible. The antibodies used are shown in Table 2.

### Statistical analysis

All data were analyzed with GraphPad Prism version 8.03 (GraphPad Software, La Jolla, CA) using the paired t-test. Values of *p* < 0.05 (95% confidence interval) were considered to be statistically significant.

### Human ethics statement

All methodology followed research integrity covering rules, regulations and best practices in accordance with relevant guidelines and regulations. Tissue samples were collected after informed written consent and approval of the Medical Ethics Committee (METc) of the University Medical Center Groningen (2007/067).

### Ethics approval and consent to participate

Obtained.

### Consent for publication

Not applicable.

## Results

### Dupuytren nodular fibroblasts show upregulation of fibrosis-related genes at mRNA level after TGF-β1 stimulation

**Figure 2:**
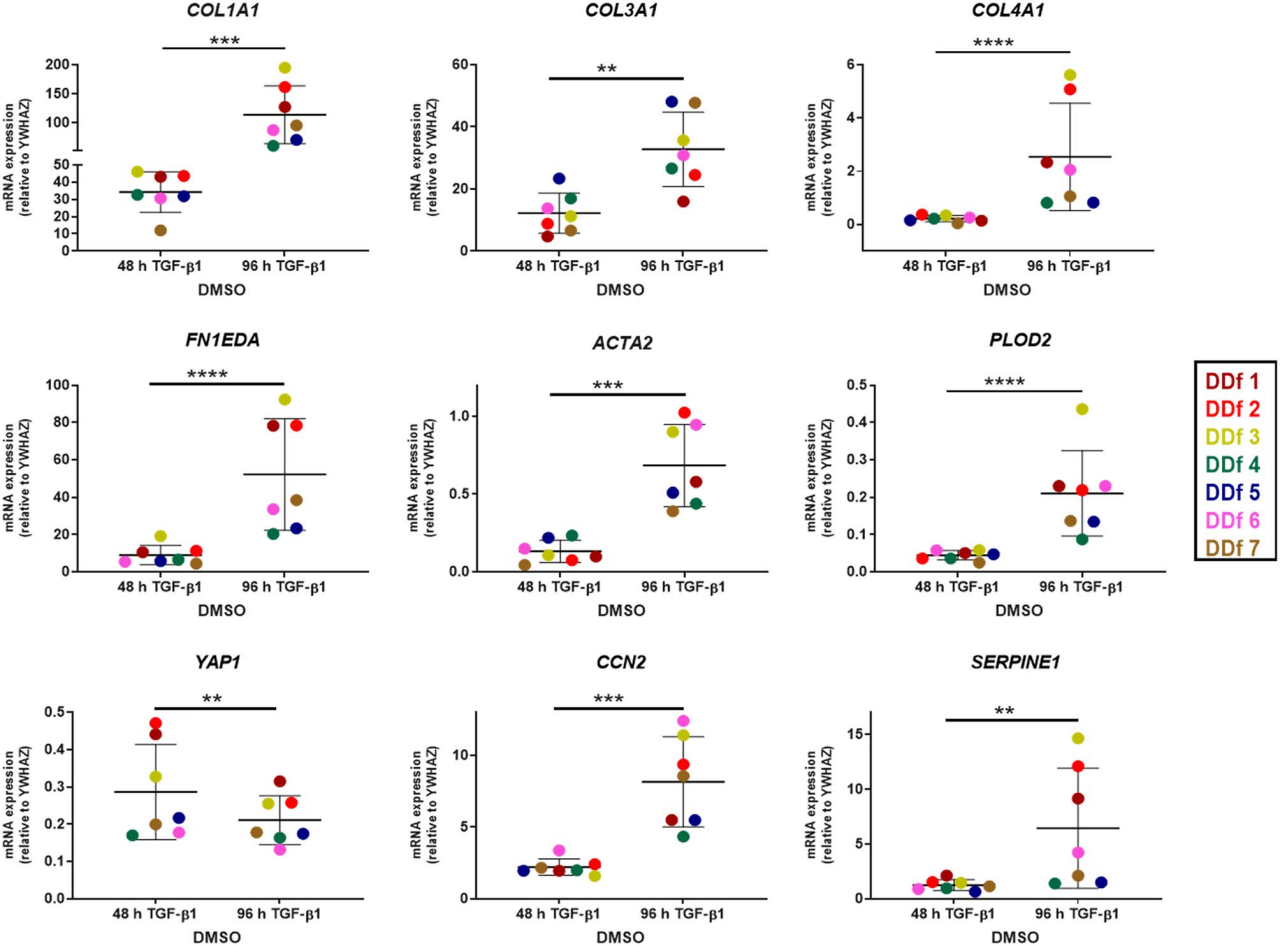
Effect of 48 h and 96 h TGF-β1 stimulation (5 ng/ml) of DD nodule-derived fibroblasts (n = 7) in the absence of verteporfin. mRNA expression of COL1A1, COL3A1, COL4A1, FN1EDA, ACTA2, PLOD2, YAP1, CCN2 and SERPINE1 relative to YWHAZ.

We performed quantitative PCR for *COL1A1*, *COL3A1*, *COL4A1* and *FN1EDA*, being a set of extracellular matrix genes that are upregulated in fibrotic pathologies. Other fibrosis-related genes that we measured are *SERPINE1* (an inhibitor for plasmin-mediated MMP activation), *PLOD2* (an enzyme involved in collagen cross-linking), *YAP1* and its downstream target *CCN2*. Finally, we measured *ACTA2*, encoding for alfa-smooth muscle actin, that form stress fibers in myofibroblasts. With the exception of *YAP1*, we observed significant upregulation of all fibrosis-related genes after 96 h TGF-β1 stimulation in comparison with 48 h TGF-β1 stimulation (Fig. 2). Its shows that 48 h of exposure to 5 ng/ml TGF-β1 is not enough to obtain full activation of the fibroblasts. In fact, we observe on average a 3.3-fold increase of mRNA levels of *COL1A1* (*p* = 0.0006), 2.7-fold increase of *COL3A*1 (*p* = 0.0013), 11.4-fold increase of *COL4A1* (*p* =  < 0.0001), 5.8-fold increase for *FN1EDA* (*p* =  < 0.0001), 3.7-fold increase for *CCN2* (*p* = 0.0001), 5.1-fold increase for *ACTA2* (*p* = 0.0008), 4.7-fold increase for *PLOD2* (*p* =  < 0.0001), 5.2-fold change for *SERPINE1* (*p* = 0.0031). We observed a significant downregulation of the *YAP1* gene, showing a 1.4-fold change (*p* = 0.0082) (Fig. 2). Our results clearly show increased mRNA levels of fibrotic markers in nodule fibroblasts with prolonged TGF-β1 stimulation time. Furthermore, we observed different delta upregulation values after 96 h TGF-β1 exposure between the patients, which seems to indicate a high heterogeneity between patients suffering from DD.

### VP treatment decreases gene expression of fibrosis-related genes at mRNA levels in Dupuytren nodular fibroblasts

Next, we investigated the effect of verteporfin in the presence or absence of TGF-β1 on the expression of the genes mentioned in the previous section (*COL1A1*, *COL3A1*, *COL4A1*, *FN1EDA*, *SERPINE1*, *PLOD2*, *YAP1*, *CCN2* and *ACTA2*) in nodule fibroblasts.

**Figure 3:**
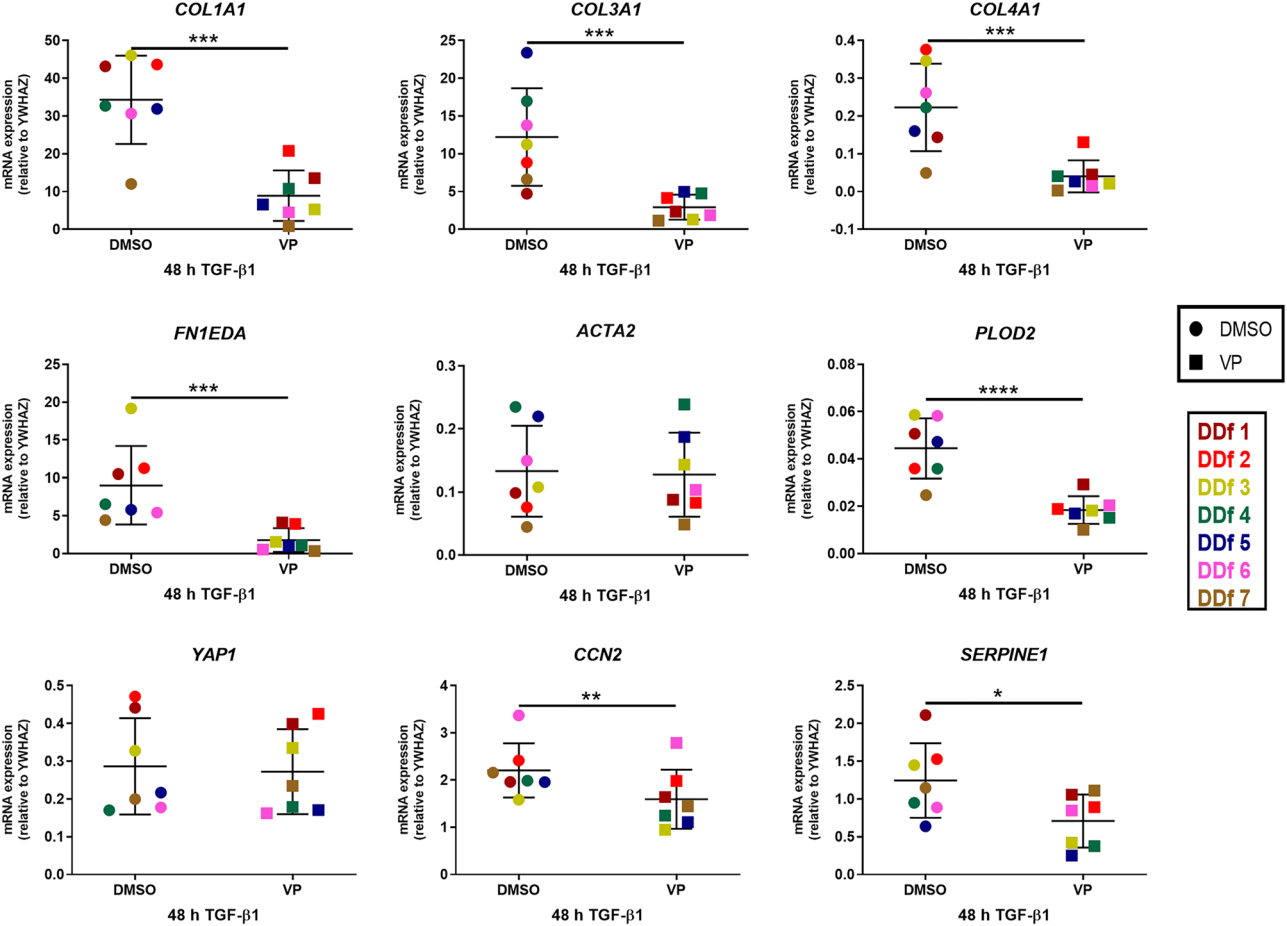
Effect of VP on DD nodule-derived fibroblasts exposed to 48 h of TGFβ1 (n = 7). mRNA expression of COL1A1, COL3A1, COL4A1, FN1EDA, ACTA2, PLOD2, YAP1, CCN2 and SERPINE1 relative to YWHAZ after 48 h of treatment with VP (with DMSO as control) in the absence of TGF-β1.

**Figure 4:**
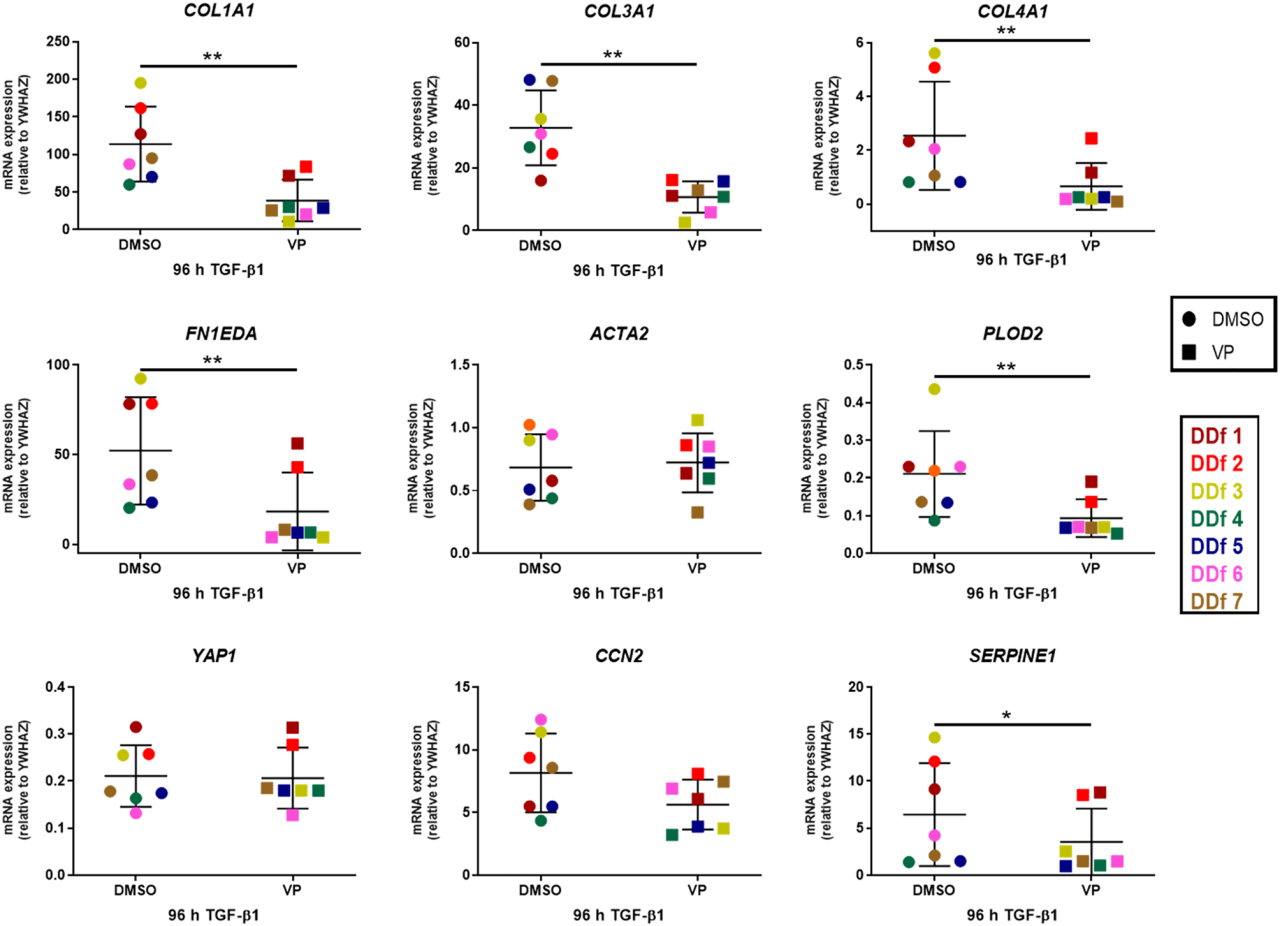
Effect of VP on DD nodule-derived fibroblasts (n = 7). mRNA expression of COL1A1, COL3A1, COL4A1, FN1EDA, ACTA2, PLOD2, YAP1, CCN2 and SERPENI1 relative to YWHAZ; the cells were treated with 5 ng/ml of TGF-β1 stimulation for 96 h including a 48 h treatment with VP (with DMSO as control).

We found that VP decreases significantly the expression levels of the 4 genes encoding for ECM proteins (*COL1A1*, *COL3A1*, *COL4A1*, *FN1EDA*), both in the presence or absence of TGF-β1. In the group without the presence of TGF-β1, we observed a decrease in 7 of the 9 genes tested (*COL1A1*, *COL3A1*, *COL4A1*, *FN1EDA*, *PLOD2*, *CCN2*, and *SERPINE1*) with values ranging from 1.4 to 5.5 (Fig. 3). In the group that was exposed to an additional 48 h with TGF-β1, we observed a decrease in 6 of the 9 genes tested (*COL1A1*, *COL3A1*, *COL4A1*, *FN1EDA*, *PLOD2*, and SERPINE1) with values ranging from 1. 8 to 3.9 (Fig. 4).

We do not observe a decrease in *ACTA2* and *YAP1* gene expression when exposed to VP, regardless whether TGF-β1 was present or not. A significant decrease of *CCN2* gene expression was seen in the group cultured in the absence of TGF-β1 stimulation. No such significance could be shown in the group cultured in the presence of TGF-β1 stimulation, although a trend in decreasing levels can be observed. Detailed foldchanges in gene expression are presented in Table 3.

**Table 3:** Fold-changes in mRNA gene expression between control (DMSO) and VP-treated cells. Ratio of gene expression decrease after 48 h treatment with DMSO vs VP. ↓: Indicates decrease. @ Indicates that the decrease is statistically non-significant, although a trend is present.

GENE	Fold change DMSO/VP			
	− TGF-β1	*p* value	+ TGF-β1	*p* value
*COL1A1*	↓3.9	0.0007	↓2.9	0.0078
*COL3A1*	↓4.2	0.0005	↓3.1	0.0067
*COL4A1*	↓5.5	0.0006	↓3.9	0.0044
*ACTA2*	1.0	0.7743	0.9	0.4641
*PLOD2*	↓2.4	< 0.0001	↓2.2	0.0085
*FN1EDA*	↓5.0	0.0003	↓2.8	0.0072
*YAP1*	1.0	0.4242	1.0	0.7261
*CCN2*	↓1.4	0.0013	↓1.5@	0.0514
*SERPINE1*	↓1.8	0.0108	↓1.8	0.0332

### VP affects the translation and morphology of hallmark proteins in fibrosis

**Figure 5:**
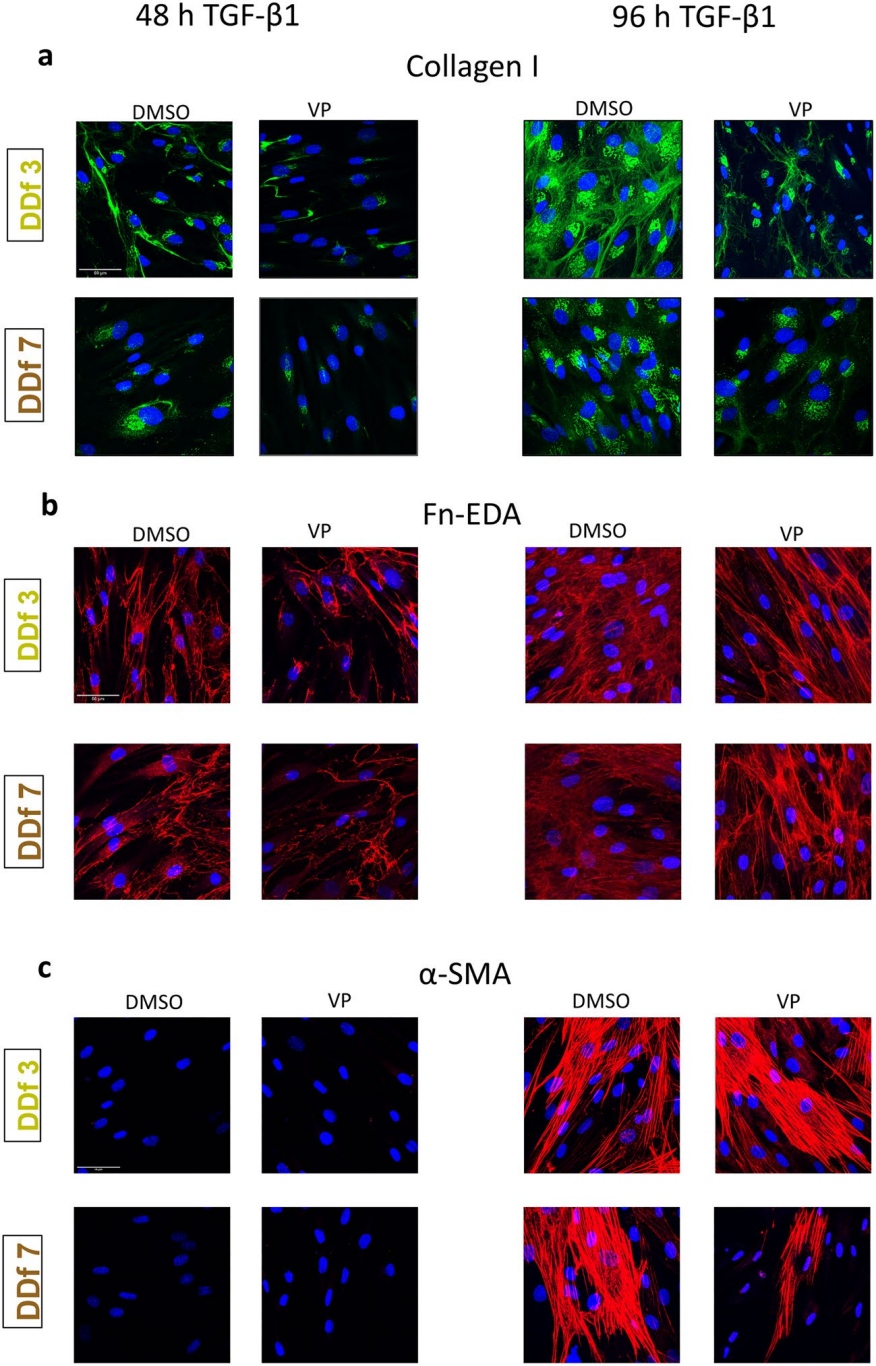
Immunofluorescence of hallmark proteins in fibrosis. (**a**) Synthesis and deposition of collagen type I after 48 and 96 h TGF-β1 stimulation in the presence or absence of VP. Immunofluorescence of intracellular and extracellular collagen type I. (**b**) Deposition and morphology of Fn-EDA after 48 and 96 h TGF-β1 stimulation in the presence or absence of VP as revealed by immunofluorescence staining. (**c**) Myofibroblast differentiation after 48 and 96 h TGF-β1 stimulation in the presence or absence of VP as revealed by immunofluorescence staining of α-SMA. Iimages are shown from donors with a high and a low expression (high: patient DDf3 and low: patient DDf7; for mRNA data see Figs. 3 and 4) (magnification 63x, scale-bar = 50 µm).

Since it is known that there can be a discrepancy between mRNA levels and protein levels, especially in the case of extracellular matrix proteins, we also stained the presence of collagen type I and fibronectin-EDA (fn-EDA) in the cell cultures. Furthermore, we stained the α-SMA stress fibers (the marker of mature myofibroblasts) in the cells themselves. Collagen type I shows a more intense staining with increasing TGF-β1 incubation time (48 vs. 96 h), both intracellularly and as extracellular deposits (Fig. 5a). VP significantly decreased the amount of stained collagen, both in the presence or absence of TGF-β1. Fn-EDA also showed a more intense staining at 96 h TGF-β1 incubation time. As in the case with collagen, the presence of VP decreased the amount of stained fn-EDA (Fig. 5b). In addition, changes in morphology of the deposited fn-EDA are seen. When cells are not stimulated with TGF-β1, a reticular pattern of deposition is observed, which is shifted to a net-like deposition in the presence of TGF-β1. After treatment with VP the reticular pattern is again seen (Fig. 5b). With respect to α-SMA, hardly any α-SMA positive cells were seen at 48 h TGF-β1 stimulation, while clearly increasing numbers were seen at 96 h (Fig. 5c). The presence of VP resulted in a decrease in α-SMA positive cells in a subset of patients only.

**Figure 6:**
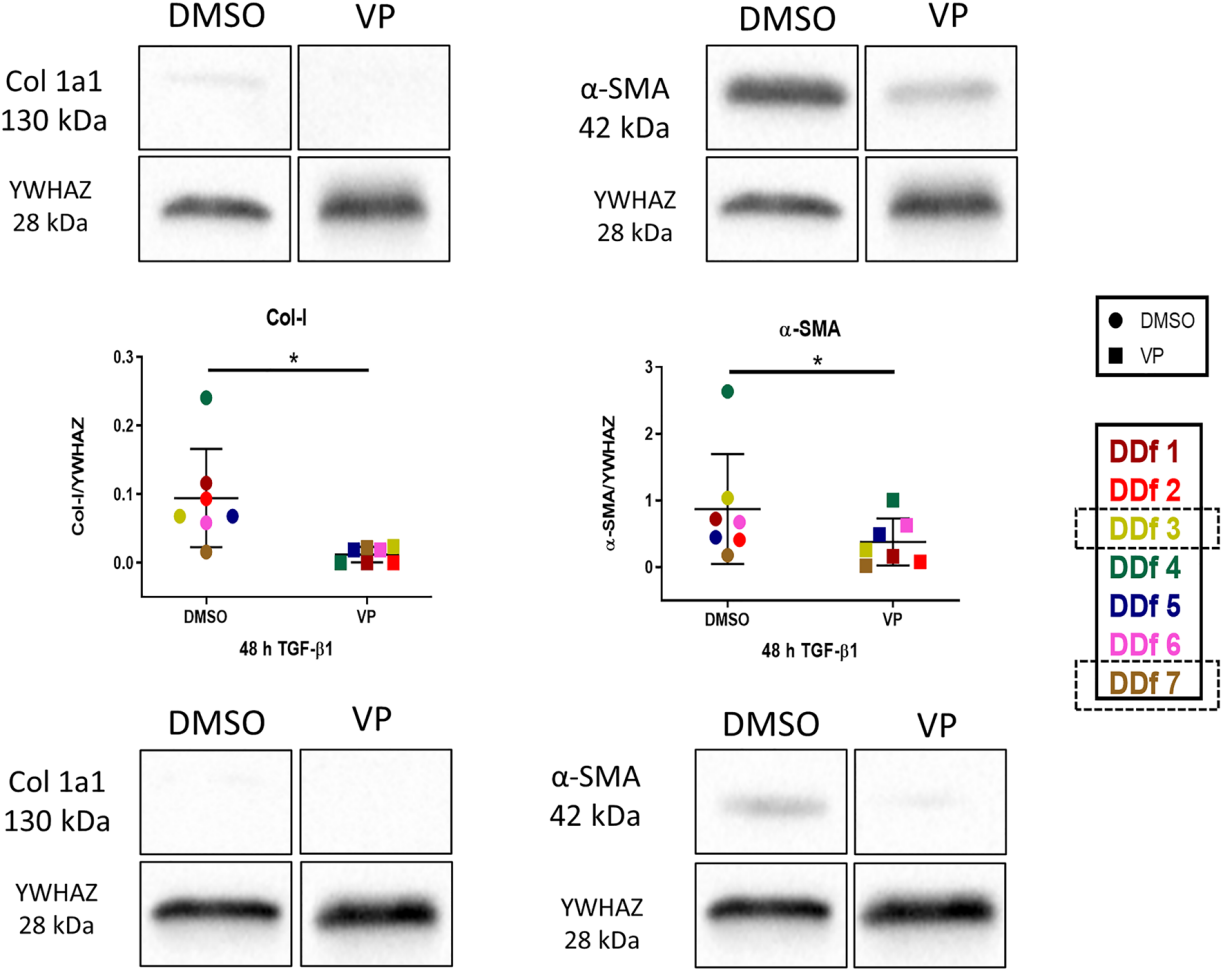
Western blot analysis and quantification of Col 1a1 and α-SMA in DD nodule-derived fibroblasts after stimulation for 48 h with TGF-β1 followed by 48 h in the presence or absence of VP. Images from donors with high and low expression (high: patient DDf3 and low: patient DDf7). Original blots are presented in Supplementary Fig. 1 (S1).

**Figure 7:**
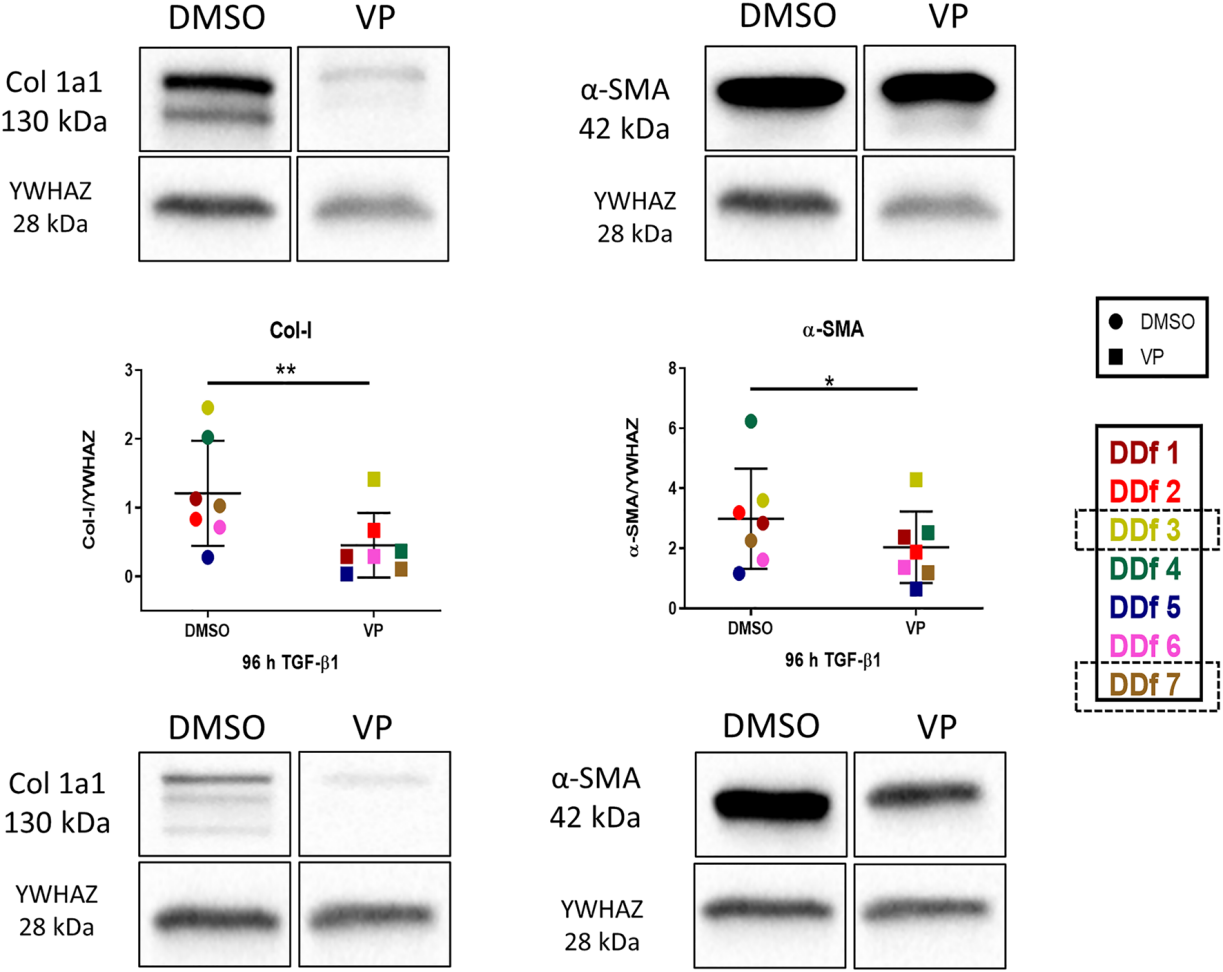
Western blot analysis and quantification of Col 1a1 and α-SMA in DD nodule-derived fibroblasts after stimulation for 96 h with TGF-β1 but in the last 48 h in the presence or absence of VP. Images from donors with high and low expression (high: patient DDf3 and low: patient DDf7). Original blots are presented in Supplementary Fig. 2 (S2).

To strengthen the results as obtained by optical analyses, we next quantified protein levels of collagen type I by Western blot. It is important to note that the protein samples were obtained from cell lysates, so we can only quantify intracellular collagen and not collagen deposited extracellularly, which is the case for collagen type I. As expected, collagen type I levels are rather low when stimulated with TGF-β1 for 48 h only; it became even lower in the presence of VP (Fig. 6). More collagen was observed when cells were stimulated for 96 h, and also here a significant decrease is seen of collagen type I in the presence of VP (Fig. 7). In the case of the myofibroblast marker α-SMA, a major increase is seen with prolonged TGF-β1 incubation times (48 and 96 h), and in both cases α-SMA protein levels were lower in the presence of VP (Fig. 7). This decrease in protein level VP which was not clearly evident by immunostaining of the cells themselves (compare Fig. 5c with Fig. 7).

### VP affects protein levels of YAP, SMAD2 and SMAD3

**Figure 8:**
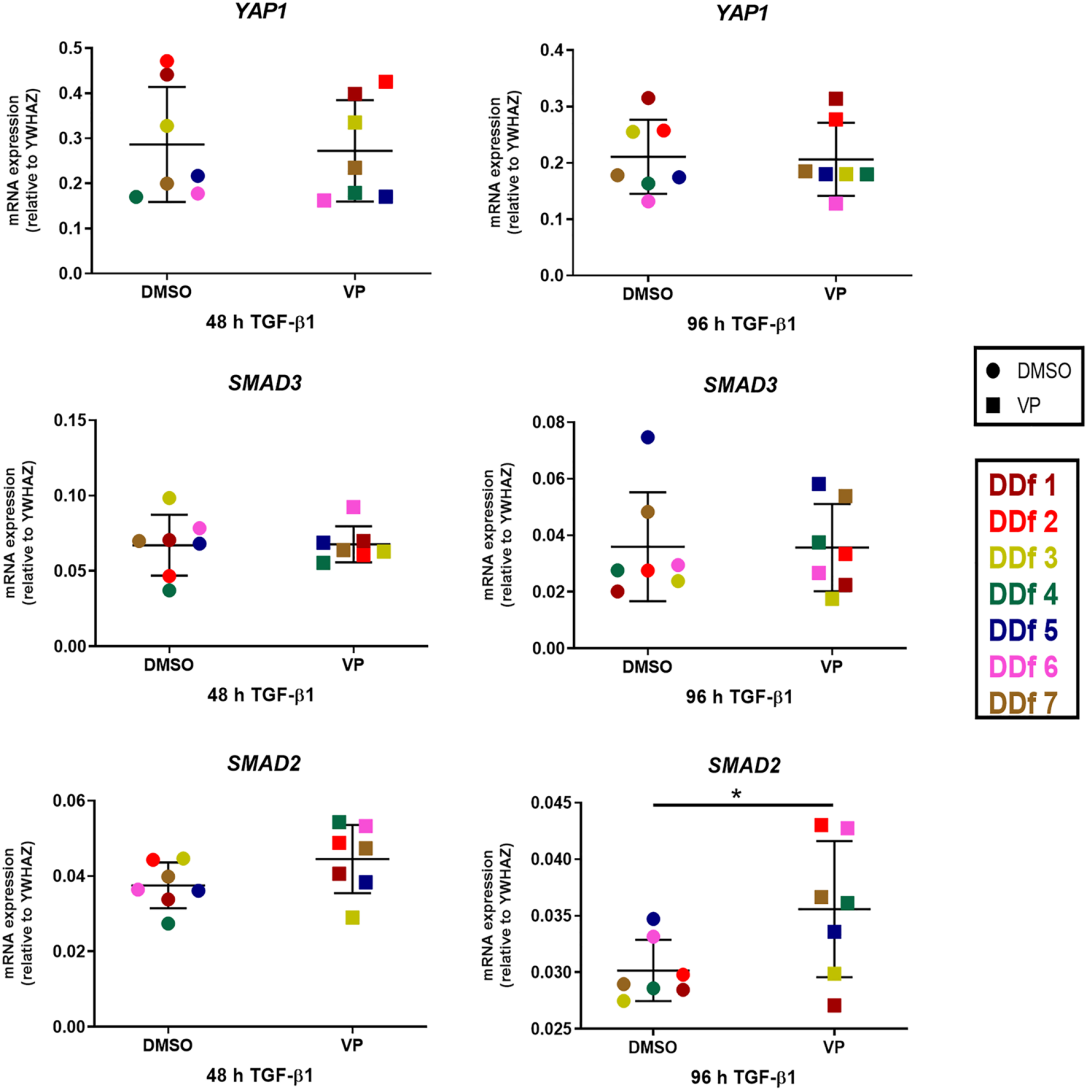
Effect of VP treatment on TGF-β/Hippo pathway downstream effectors. Relative mRNA expression of YAP1, SMAD2 and SMAD3 after stimulation with TGF-β1 for 48 h followed by 48 h in the presence or absence with VP, or after stimulation with TGF-β1 for 96 h but in the last 48 h in the presence or absence of VP.

Since we observed that VP downregulates major fibrogenic genes, we further determined the effect of VP on the downstream effectors of the TGF-β and Hippo pathway. We studied gene expression by quantitative PCR and protein levels by Western blot of total *YAP1* and *SMAD2* and *SMAD3*. No changes were observed in gene expression levels of *YAP1* and *SMAD3* by VP, neither with or without the presence of TGF-β1 for 48 or 96 h. In contrast, we observed an upregulation of *SMAD2* gene expression after VP treatment in the group under continuous (96 h) TGF-β1 stimulation (Fig. 8).

**Figure 9:**
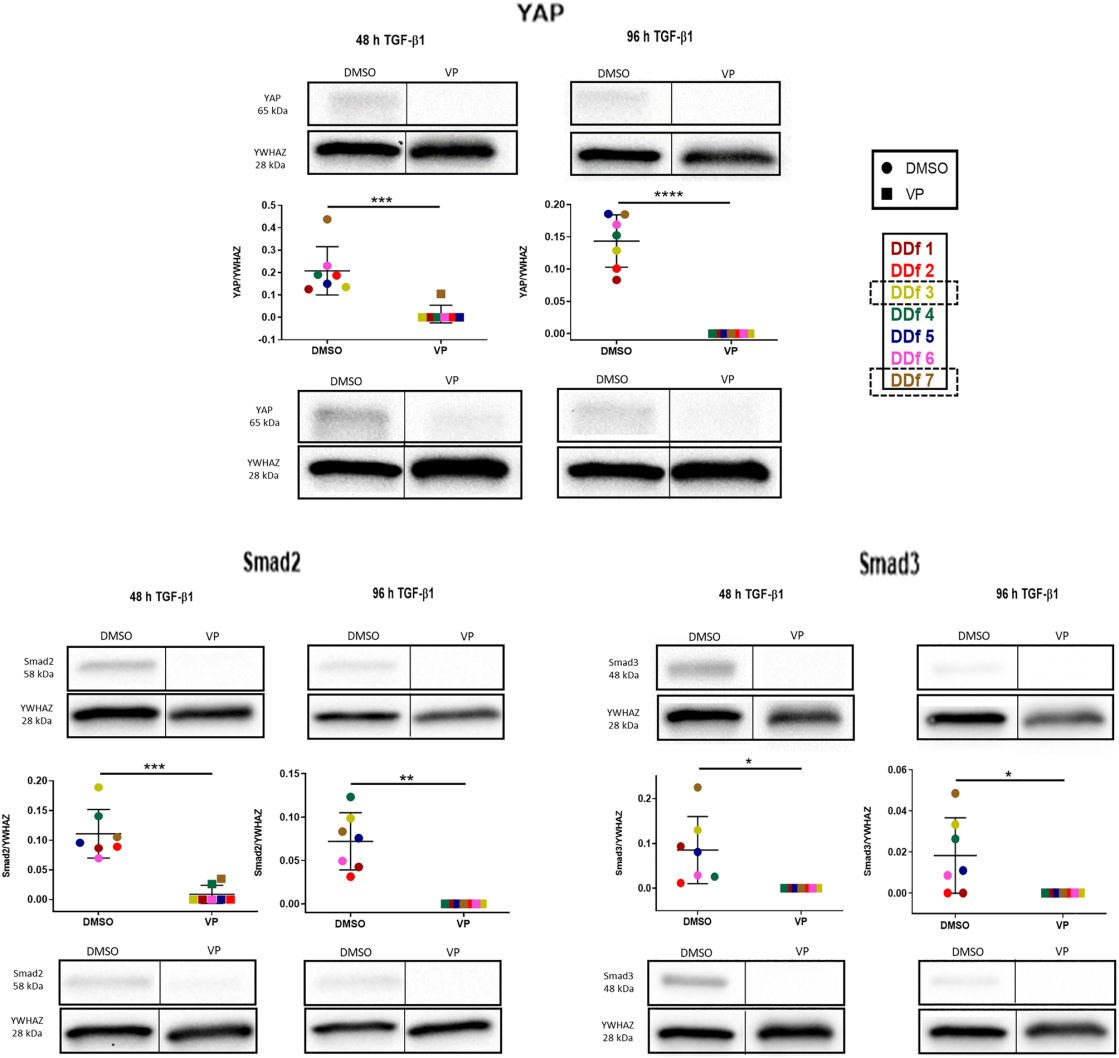
Effect of VP treatment on TGF-β/Hippo pathway downstream effectors. Western blot analysis and quantification of YAP, Smad2, and Smad3, after stimulation with TGF-β1 for 48 h followed by 48 h in the presence or absence with VP, or after stimulation with TGF-β1 for 96 h but in the last 48 h in the presence or absence of VP. Images are shown from donors with a high and low expression (high: patient DDf3 and low: patient DDf7). Original blots are presented in Supplementary Figs. 3 and 4 (S3 and S4).

On protein expression level, a mild decrease of protein level is seen for all three proteins (YAP1, Smad2 and Smad3) studied as a result of TGF-β1 stimulation. Remarkably, all three proteins become essentially undetectable after VP treatment (Fig. 9).

## Discussion

DD is a chronic, fibroproliferative disorder of the hand fascia, which results in impairment of the extension of fingers and therefore hinders a normal use of the hand. Treatments for DD in early stages unfortunately show lack of effectiveness, while the management of the final stage is invasive and often results in disease recurrence^15^. Therefore, the need for a treatment that at least prevents the progression of the disease is high. Here we show with in vitro cell cultures of DD nodular fibroblasts that VP is not only able to halt the activation of fibroblasts into myofibroblasts, but is also able to reverse the activation status of the cells.

Differentiation of fibroblasts into myofibroblasts is a relatively slow process. In this study, we first cultured DD nodular fibroblasts in the presence of TGF-β1 for 48 h. After this stimulation period, we subsequently treated the fibroblasts for 48 h with or without verteporfin under two regimes: with or without TGF-β1. In the absence of VP, we observed that in the additional 48 h a further acceleration of the activation status takes place in the presence of TGF-β1. In this additional time period, an increase is seen in mRNA levels of *COL1A1*, *COL3A1*, *COL4A1*, *FN1EDA*, *PLOD2*, *ACTA2*, *CCN2* and *SERPINE1*, which is substantiated by the increased protein levels of collagen type I, fn-EDA and α-SMA. In this experimental setup, one is able to investigate^1^ whether VP is able to push back the achieved activation status of the first stimulation period (namely the experiment with VP in the absence of TGF-β1), and^2^ whether VP is able to halt an ongoing activation in cells that are already triggered to become myofibroblasts (namely the experiment with VP in the presence of TGF-β1).

Our experiments show that VP has strong anti-fibrotic properties: it not only pushes back the activation status of fibroblasts that are exposed for 48 h to TGF-β1, but it also halts an ongoing activation of cells that are in the process of becoming myofibroblasts. In both experimental settings mRNA levels of *COL1A1*, *COL3A1*, *COL4A1*, *FN1EDA*, *PLOD2*, *CCN2* and *SERPINE1* are significantly decreased, and this is also observed in the protein levels of collagen type I. We observed in addition changes in the morphology of the deposited fn-EDA, namely from a mesh-like deposition to a reticular network in the presence of VP (the other genes were not investigated with regard to protein levels). Keeping in mind that the hallmark of fibrosis (and DD) is an unwanted accumulation of collagen (especially collagen type I), this is quite promising.

The decrease of *COL1A1* gene expression after VP treatment has also been found in in vivo preclinical models of kidney and hepatic fibrosis^29,32,35^. It has also been observed in rodent and human in vitro models, namely rat hepatic stellate cells, angiotensin II-stimulated primary human ventricular fibroblasts, and TGF-β1 stimulated dermal fibroblasts^33,36,39^. Moreover, our results correlate with data obtained from myofibroblasts isolated from other pathological tissues, namely plaque tissue from Peyronie’s disease and forearm skin of patients with diffuse subset scleroderma, where expression of *COL1A1* is also decreased after VP treatment^37,39^. As is the case with mRNA, a decrease in protein expression of collagen type I has also been reported^18,29–32,34,]^^35^.

The expression of a variety of profibrotic genes is activated by nuclear YAP and Smad2/3^18,29,30,]^^31,32,35,36,38,40^. We therefore measured mRNA and protein levels of these three molecules. We found no effect of VP on mRNA levels of these three genes when fibroblasts are stimulated for 48 h with TGF-β1. The same was the case with *YAP1* and *SMAD3* in fibroblasts stimulated for 96 h with TGF-β1, whereas *SMAD2* was slightly upregulated. However, in all cases the protein levels of YAP1, Smad2 and Smad3 were dramatically decreased in the presence of VP, being an explanation why VP is able to attenuate the expression of a large variety of important pro-fibrotic molecules.

The accumulation of collagen in fibrotic diseases (such as in DD) is due to a disbalance between collagen synthesis and collagen degradation. In fibrosis, more collagen is synthesized, and there is less collagenolytic activity. *SERPINE1* is an inhibitor for plasmin-mediated MMP activation^41,42^. VP not only attenuated the synthesis of collagen, it also attenuated *SERPINE1* levels, thus facilitating plasmin-mediated MMP activation (and consequently collagen breakdown). *PLOD2* encodes for lysyl hydroxylase 2, an enzyme involved in the formation of a specific cross-link type of collagen, making collagen more resistant towards collagenases^7,43,44^. We found that VP also decreases mRNA levels of *PLOD2*, thus making the deposited collagen more susceptible towards collagenases.

It is well established, that myofibroblasts are at the heart of fibrosis: these are the cells that produce the collagen that replaces functional tissues into fibrotic tissues. Myofibroblasts are characterized by the presence of α-SMA stress fibers; these fibers are absent in fibroblasts. Interestingly, VP did not affect mRNA levels of *ACTA2* (encoding for α-SMA), only modestly affected protein levels (as revealed by Western blotting), and no change was seen in the percentage α-SMA positive cells in some of the patients. Despite this, all subjects showed a major decrease in e.g. collagen levels. Thus, although the cells would still be classified as myofibroblasts based on the presence of α-SMA stress fibers, the overall phenotypical properties of the cells are that of a fibroblast. Although a full inhibition of myofibroblasts can be achieved by e.g. using higher concentrations of VP^29,36,45^, such concentrations might not be necessary to achieve the desired outcome (i.e. inhibition of extracellular matrix accumulation).

*CCN2* (= *CTGF*) gene expression is induced by TGF-β1 stimulation and is a direct target of the YAP/TAZ-TEAD complex^46–48^. An inhibition of YAP by VP should therefore result in lower mRNA levels of *CCN2*. Our results show that VP treatment indeed decreases *CCN2* gene expression both in non-stimulated and stimulated cells, which is in line with other studies^18,29,34,37,39,45^.

In our study, VP did not influence the gene expression (mRNA level) of the myofibroblast marker *ACTA2* in 48 h or in 96 h stimulated cells. From the five in vitro studies that report a decrease in *ACTA2* gene expression, three used higher concentrations of VP^29,36,45^. We used a concentration of 0.25 µM, while the range of dose of others goes from 0.5 µM up to 1.5 µM. The other two studies^18,32^ used a lower or the same concentration as used by us. In myofibroblasts isolated from Peyronie’s tissue there is a significant decrease of *ACTA2* after the first 24 h of VP treatment, while after 48 h of treatment *ACTA2* was not significantly reduced anymore^37^. This suggests that a 0.25 μM concentration of VP can initially influence the transcription of *ACTA2*, but that this effect is diminished at longer treatment times. A decrease was also observed in TGF-β1 stimulated (but not unstimulated) rat kidney interstitial myofibroblasts after 24 h of treatment^18^. Unfortunately, there are no data regarding a longer treatment time to corroborate our hypothesis whether the decrease in mRNA is time-dependent. It should be stressed that although we did not see a decrease in mRNA levels of *ACTA2*, we do see a significant decrease of the protein on Western blots.

The mechanism of action of VP is not completely understood yet, but recent studies show its ability to inhibit TGF-β1 nuclear accumulation of the YAP/Smad2/3 complex in two unilateral ureter obstruction mouse models of renal fibrogenesis and renal tubulointerstitial fibrosis, as well as in human conjunctival fibroblasts and rat kidney interstitial fibroblasts^18,35,38,40^. Our results confirm that VP treatment of DD nodule-derived fibroblasts decreases the total protein levels of YAP and Smad2/3 with little influence in gene expression, correlating well with the data from previous studies, further demonstrating that VP targets DD through this mechanism of action. It is important to note that YAP silencing has not shown the same strong results as VP treatment^18,26^, which suggest the involvement of additional mechanisms of action of VP. Recent studies show that VP has an effect on the Wnt pathway as it decreases levels of β-catenin in TGF-β1 stimulated hepatic stellate cells and in TGF-β2 stimulated conjunctival fibroblasts^34,38^. Interestingly, GWAS studies revealed the possible association of several components of the WNT pathway with DD, and protein studies revealed that certain elements of the WNT pathway are indeed up- or downregulated in DD^5,6,49^.

## Conclusion

Verteporfin (known under the marketing name Visudyne) is an FDA-approved drug for the treatment of macular degeneration. The route of administration is intravenously, and needs to be photo-activated in the eye by means of a laser, resulting in a selective vaso-occlusive treatment by targeting choroidal vascular abnormalities. The anti-fibrotic properties of verteporfin do not rely on photo-activation, as the molecule inhibits YAP when it is in a non-photoinduced state. It is promising that VP is able to inhibit the expression of a large set of pro-fibrotic genes, in various preclinical fibrosis models (liver, kidney), in various fibroblasts (rat, mouse, human), and in fibroblasts that display various activation states. As shown here, it is even able to reverse the activation level of fibroblasts. Furthermore, it is effective in a concentration (0.25 μM) that is at least a magnitude below its cyctotoxicity level (2 μM; see^30^). Although further studies are necessary, verteporfin seems to have potential of a broad-spectrum anti-fibrotic drug, as shown here in the context of DD nodular fibroblasts. So far, only Nintedanib and Pirfenidone are marketed as anti-fibrotic drugs, but serious side effects have been reported, and so far, the efficacy of these drugs to prevent the progression of fibrosis is rather limited^50–54^.

## Supplementary Information

Supplementary Information.

## Data Availability

The datasets used and/or analyzed during the current study are available from the corresponding author on reasonable request.

## Abbreviations

α-SMA: α-Smooth muscle actin
BSA: Bovine serum albumin
DAPI: 4′,6-Diamidine-2′-phenylindole dihydrochloride
DD: Dupuytren’s disease
DMEM: Dulbecco’s modified Eagle medium
DMSO: Dimethyl sulfoxide
ECM: Extracellular matrix
FBS: Fetal bovine serum
Fn-EDA: Fibronectin-extra domain A
GWAS: Genome-wide association study
PBS: Phosphate-buffered salt
RT: Room temperature
TBS: Tris-buffered saline
TGF-β1: Transforming growth factor β1
VP: Verteporfin
