# Supplementary material for: Verteporfin ameliorates fibrotic aspects of Dupuytren’s disease nodular fibroblasts irrespective the activation state of the cells
Source: Sci Rep. 2022 Aug 17;12:13940. doi: 10.1038/s41598-022-18116-9 (PMC9386017; doi:10.1038/s41598-022-18116-9)
Supplement: Supplementary file 1 — Supplementary Information. [file 41598_2022_18116_MOESM1_ESM.pptx]

## Slide 1
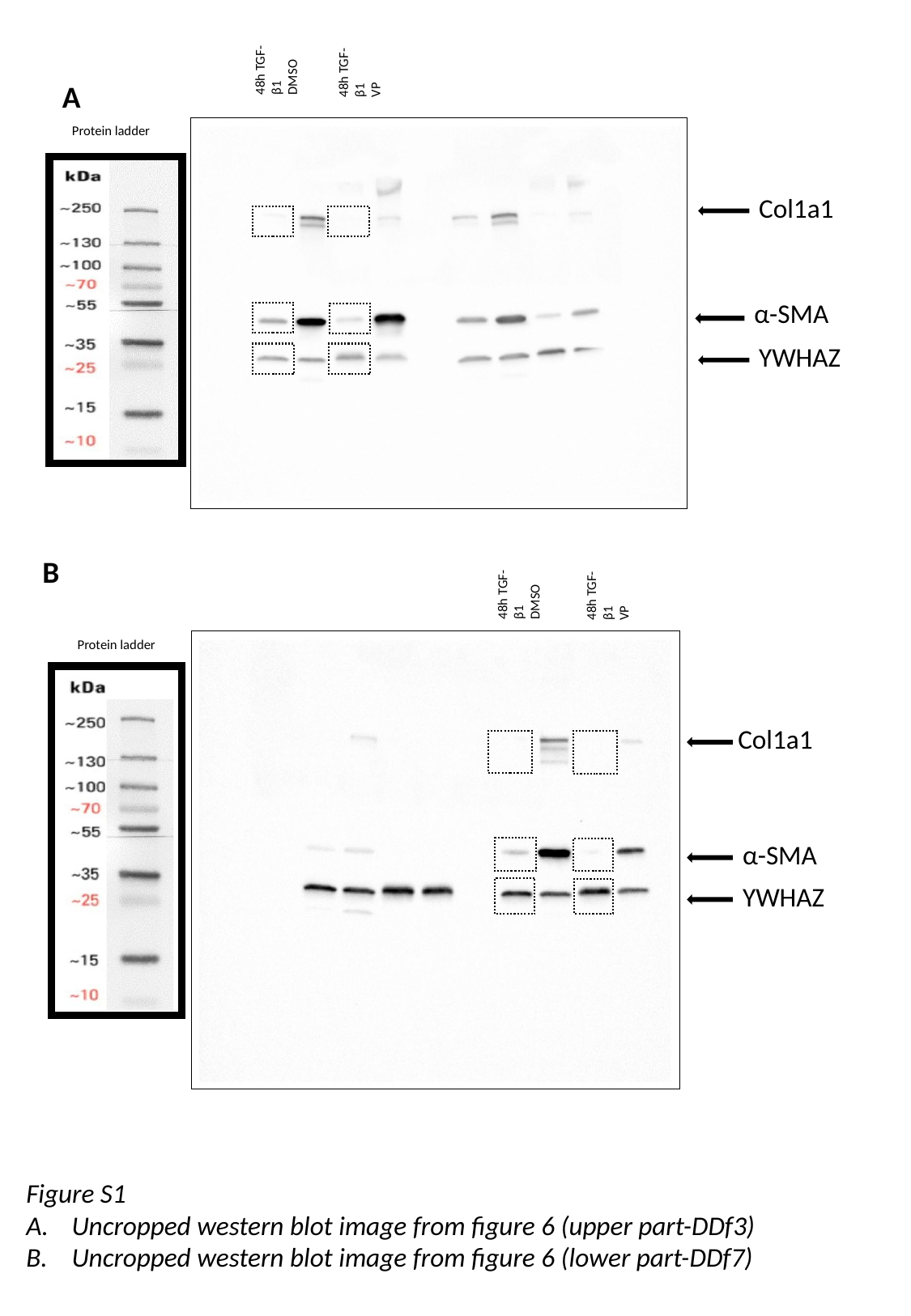

48h TGF-β1
DMSO
48h TGF-β1
VP
Col1a1
YWHAZ
α-SMA
A
Protein ladder
B
48h TGF-β1
DMSO
48h TGF-β1
VP
Col1a1
YWHAZ
α-SMA
Protein ladder
Figure S1
Uncropped western blot image from figure 6 (upper part-DDf3)
Uncropped western blot image from figure 6 (lower part-DDf7)

## Slide 2
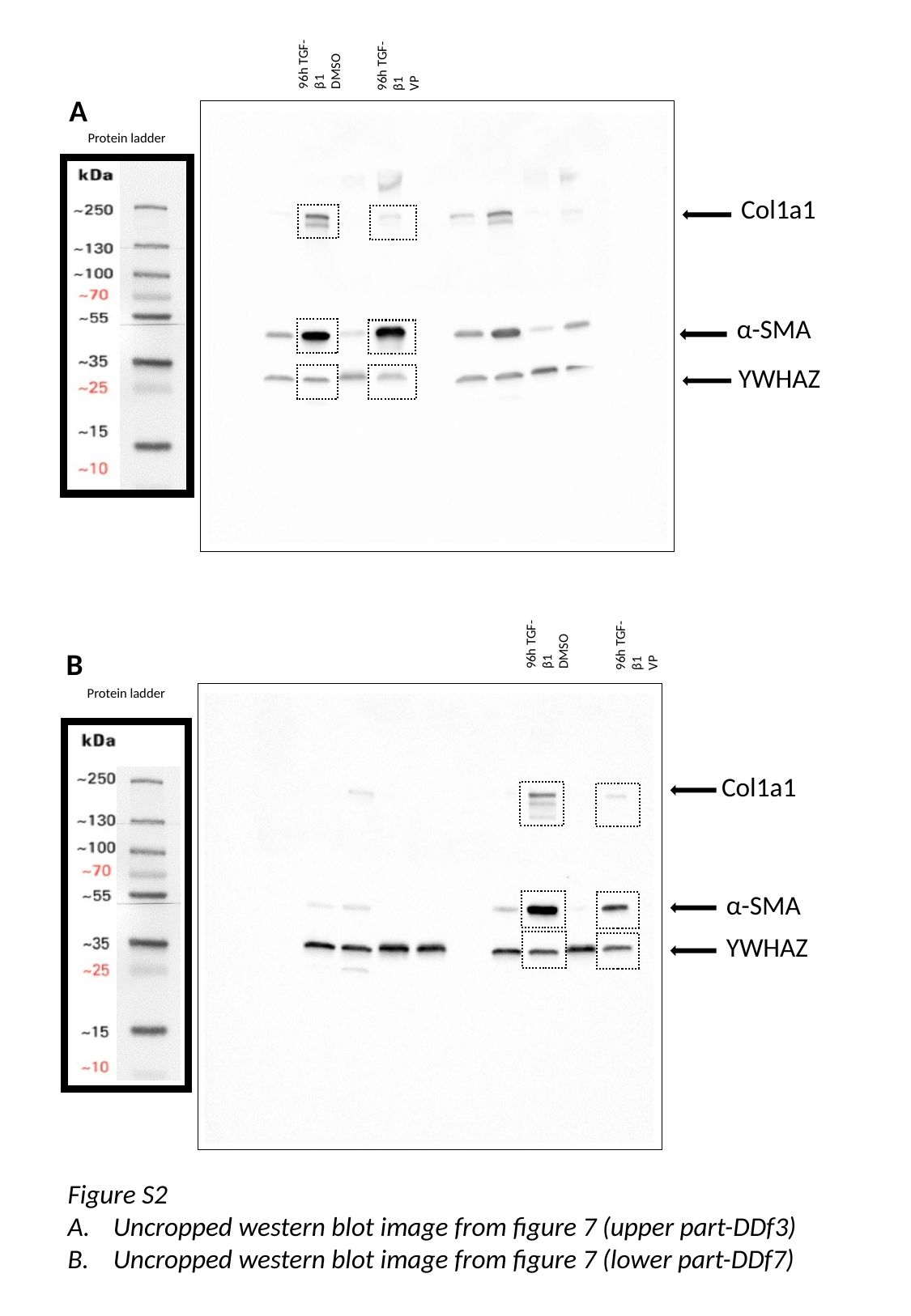

96h TGF-β1
DMSO
96h TGF-β1
VP
A
Protein ladder
Col1a1
α-SMA
YWHAZ
96h TGF-β1
DMSO
96h TGF-β1
VP
B
Protein ladder
Col1a1
α-SMA
YWHAZ
Figure S2
Uncropped western blot image from figure 7 (upper part-DDf3)
Uncropped western blot image from figure 7 (lower part-DDf7)

## Slide 3
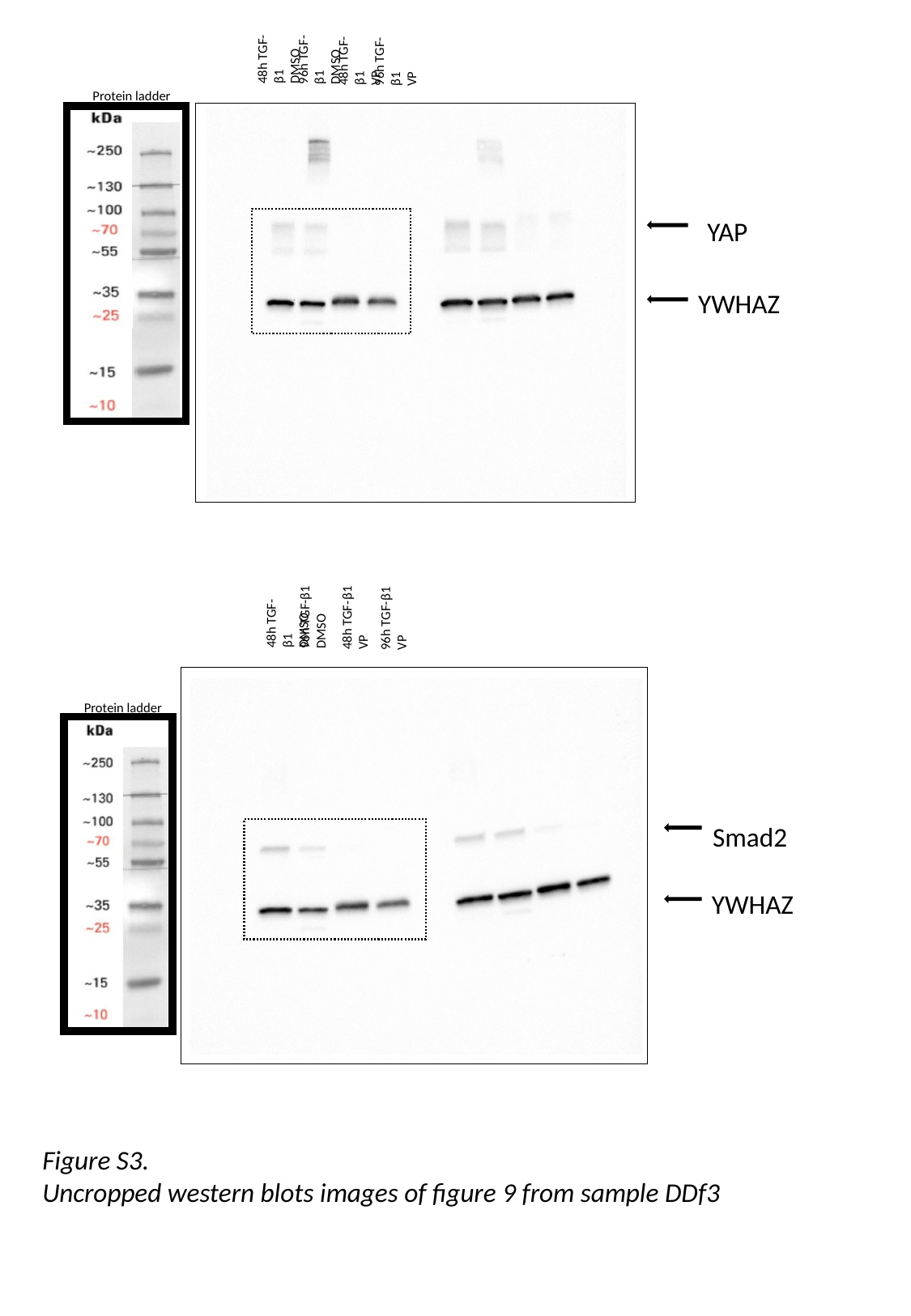

48h TGF-β1
DMSO
96h TGF-β1
DMSO
48h TGF-β1
VP
96h TGF-β1
VP
Protein ladder
YAP
YWHAZ
48h TGF-β1
VP
96h TGF-β1
DMSO
96h TGF-β1
VP
48h TGF-β1
DMSO
Protein ladder
Smad2
YWHAZ
Figure S3.
Uncropped western blots images of figure 9 from sample DDf3

## Slide 4
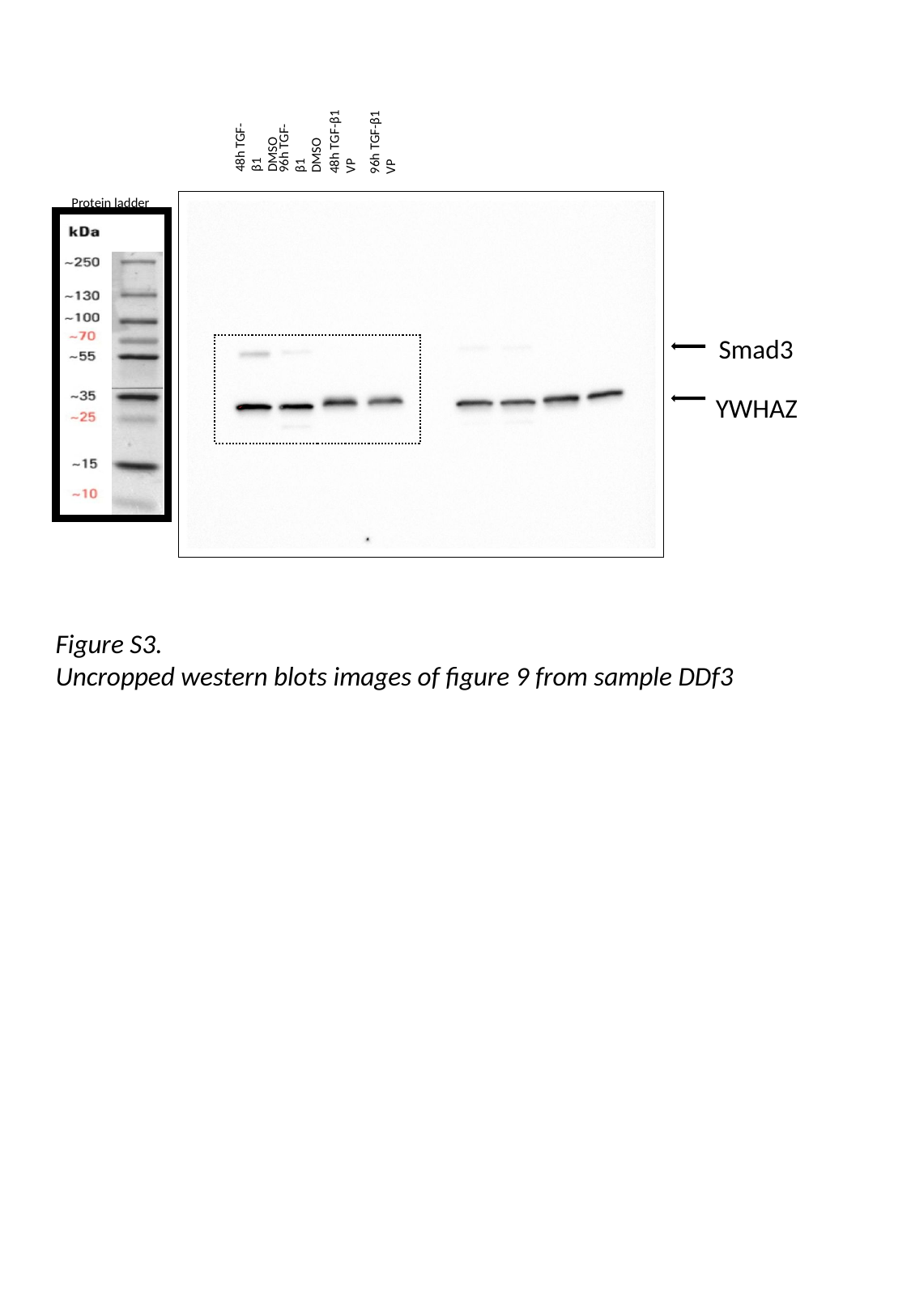

48h TGF-β1
VP
96h TGF-β1
VP
96h TGF-β1
DMSO
48h TGF-β1
DMSO
Protein ladder
Smad3
YWHAZ
Figure S3.
Uncropped western blots images of figure 9 from sample DDf3

## Slide 5
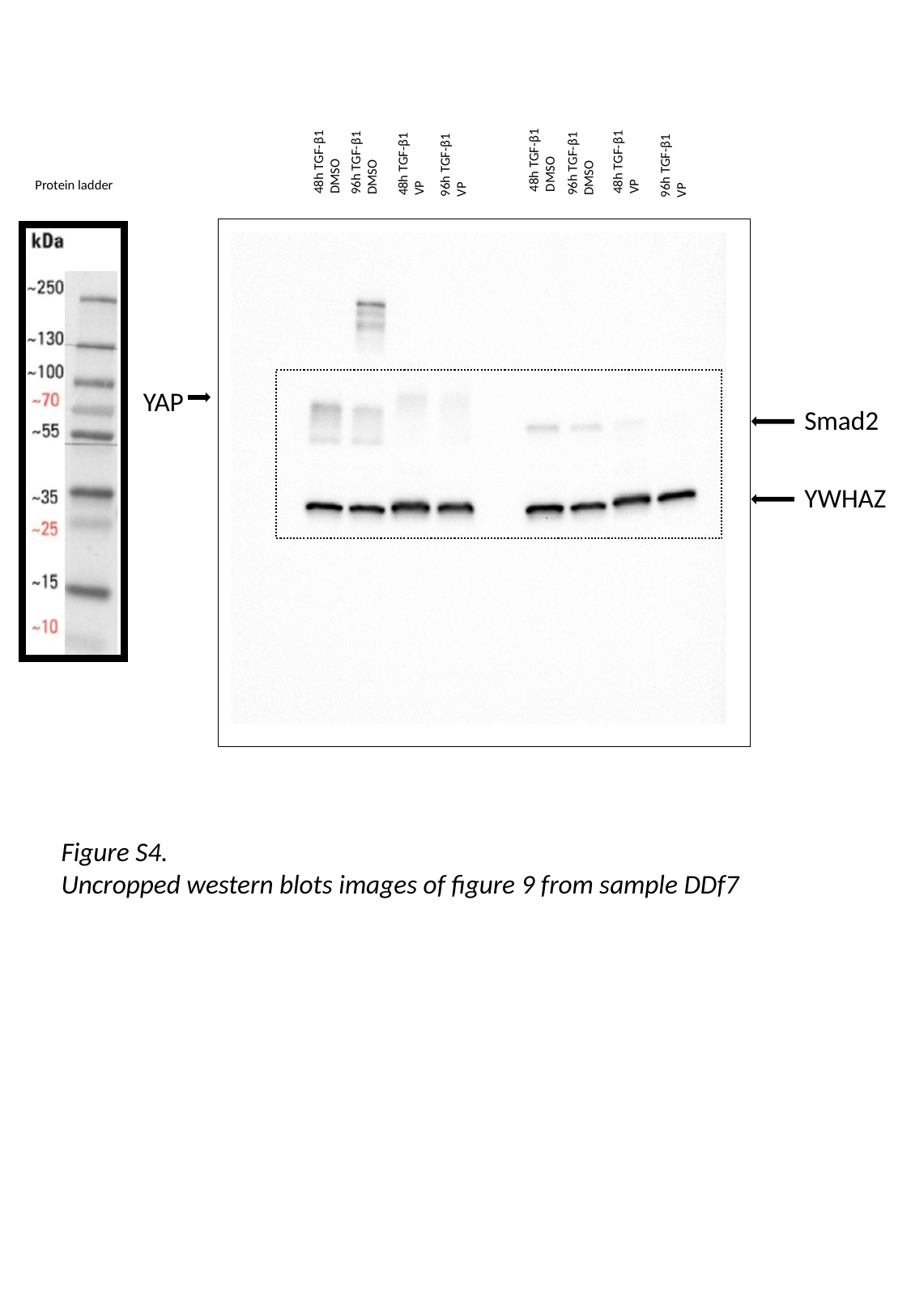

48h TGF-β1
DMSO
48h TGF-β1
DMSO
48h TGF-β1
VP
96h TGF-β1
DMSO
96h TGF-β1
DMSO
48h TGF-β1
VP
96h TGF-β1
VP
96h TGF-β1
VP
YAP
Smad2
YWHAZ
Protein ladder
Figure S4.
Uncropped western blots images of figure 9 from sample DDf7

## Slide 6
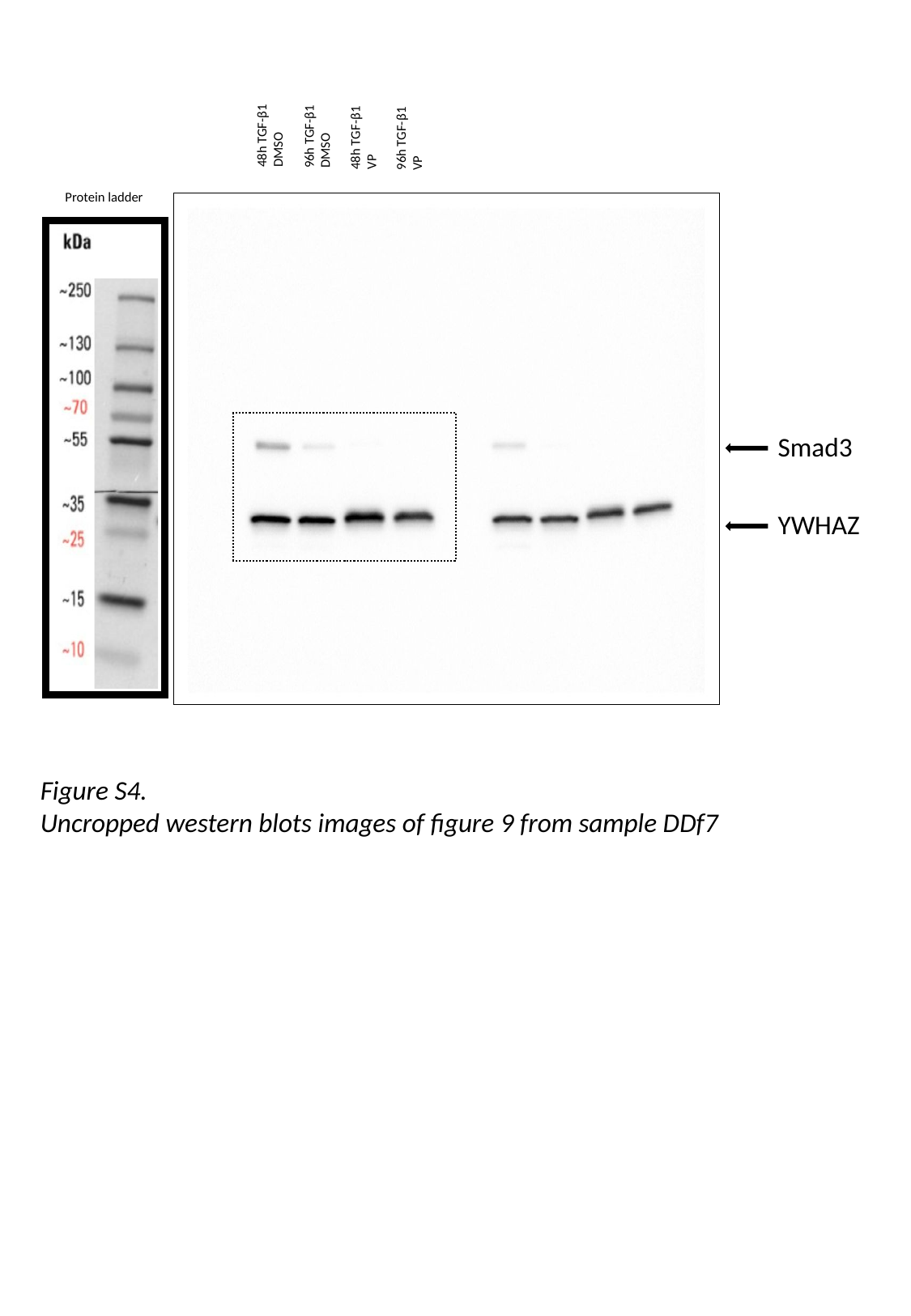

48h TGF-β1
DMSO
96h TGF-β1
DMSO
48h TGF-β1
VP
96h TGF-β1
VP
Protein ladder
Smad3
YWHAZ
Figure S4.
Uncropped western blots images of figure 9 from sample DDf7

## Slide 7
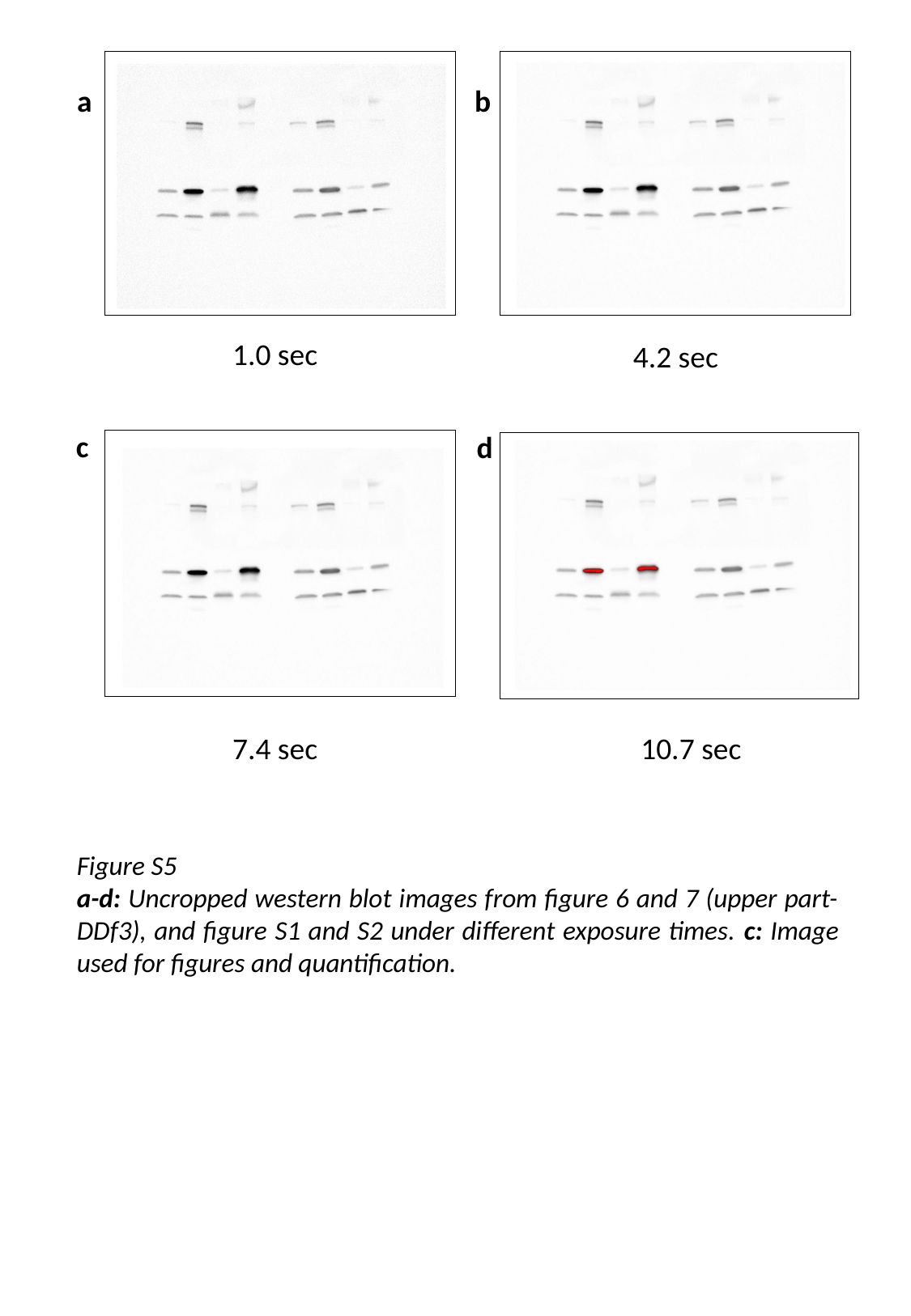

a
b
1.0 sec
4.2 sec
c
d
7.4 sec
10.7 sec
Figure S5
a-d: Uncropped western blot images from figure 6 and 7 (upper part-DDf3), and figure S1 and S2 under different exposure times. c: Image used for figures and quantification.

## Slide 8
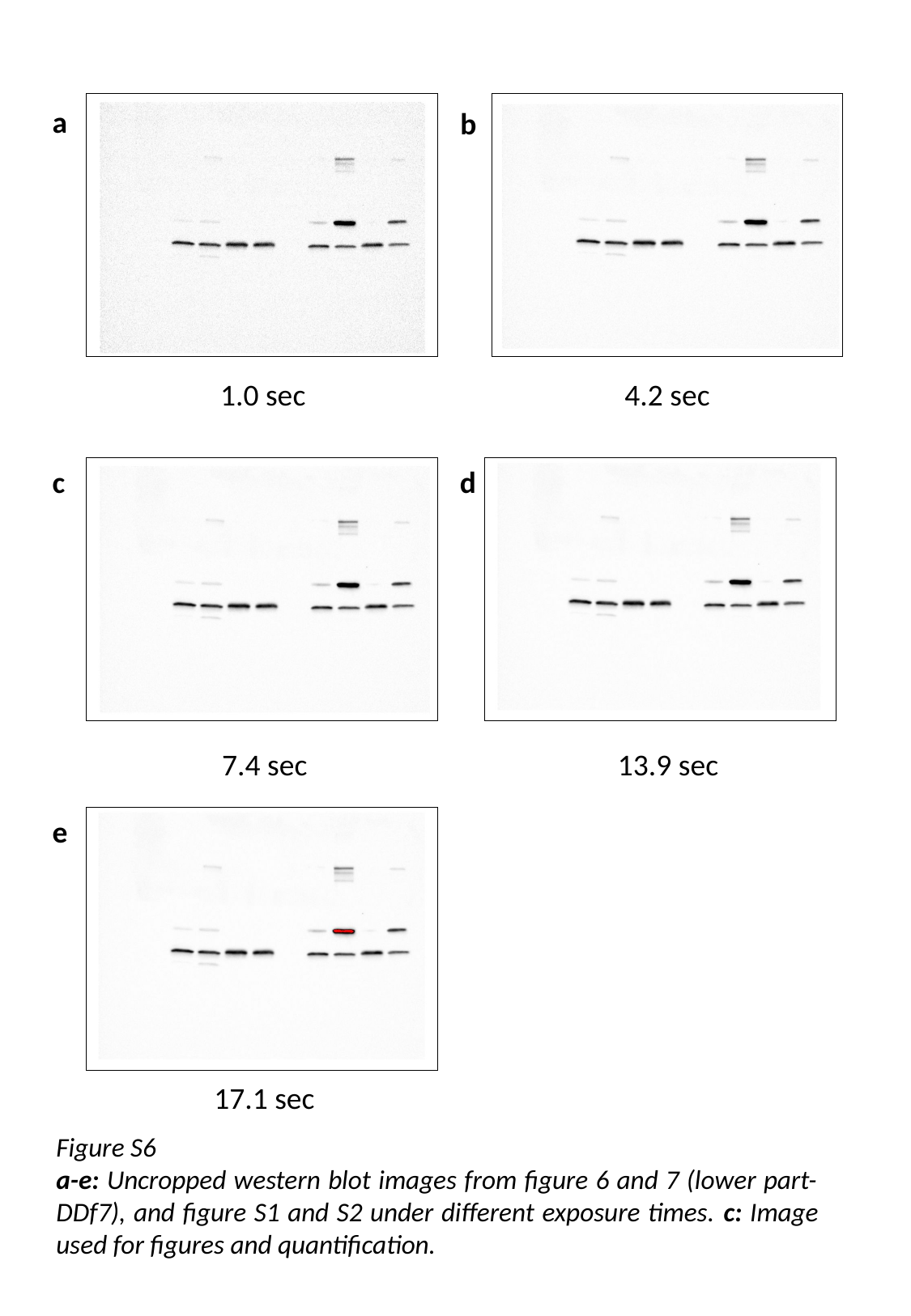

a
b
1.0 sec
4.2 sec
c
d
7.4 sec
13.9 sec
e
17.1 sec
Figure S6
a-e: Uncropped western blot images from figure 6 and 7 (lower part-DDf7), and figure S1 and S2 under different exposure times. c: Image used for figures and quantification.

## Slide 9
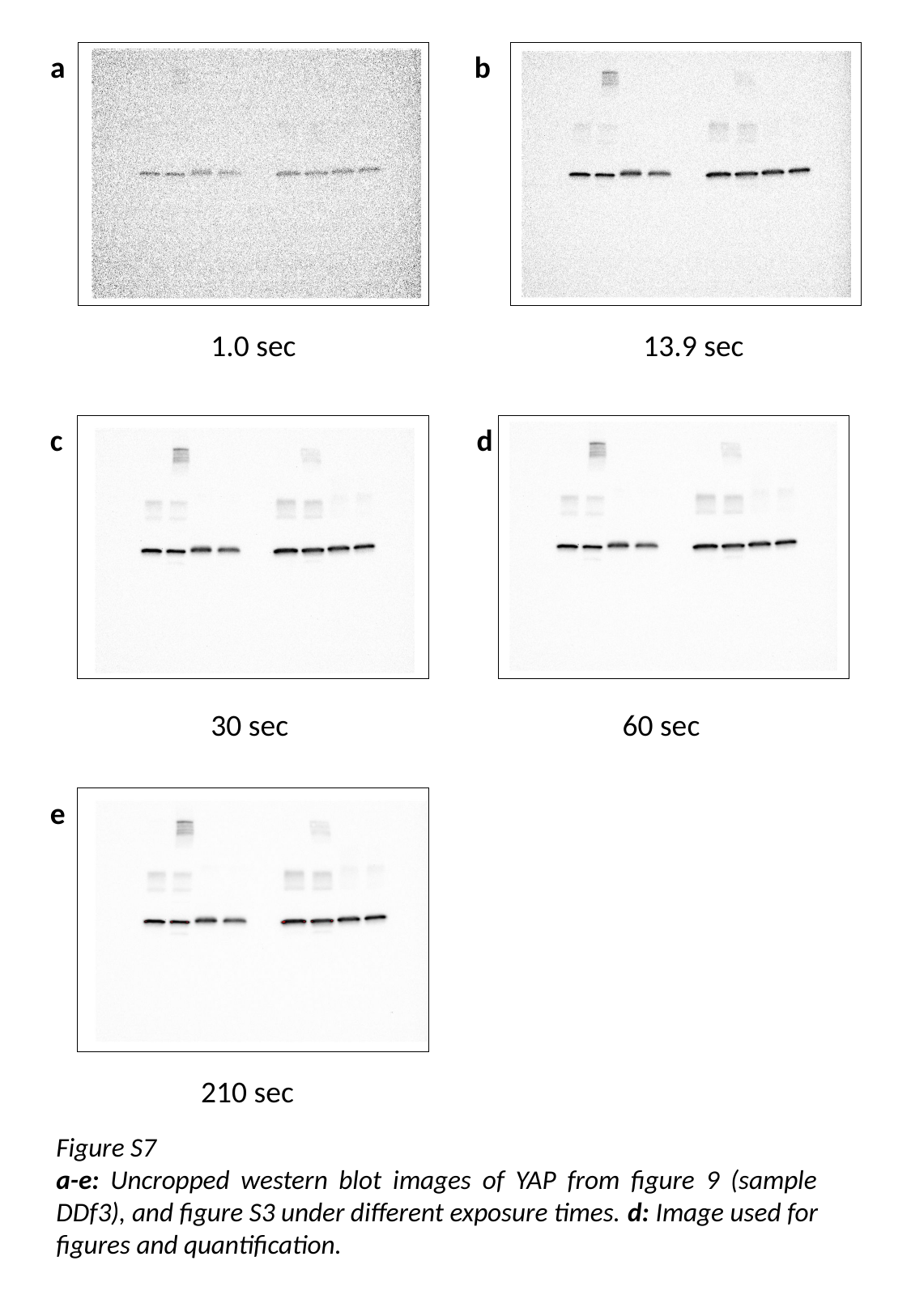

b
a
1.0 sec
13.9 sec
c
d
30 sec
60 sec
e
210 sec
Figure S7
a-e: Uncropped western blot images of YAP from figure 9 (sample DDf3), and figure S3 under different exposure times. d: Image used for figures and quantification.

## Slide 10
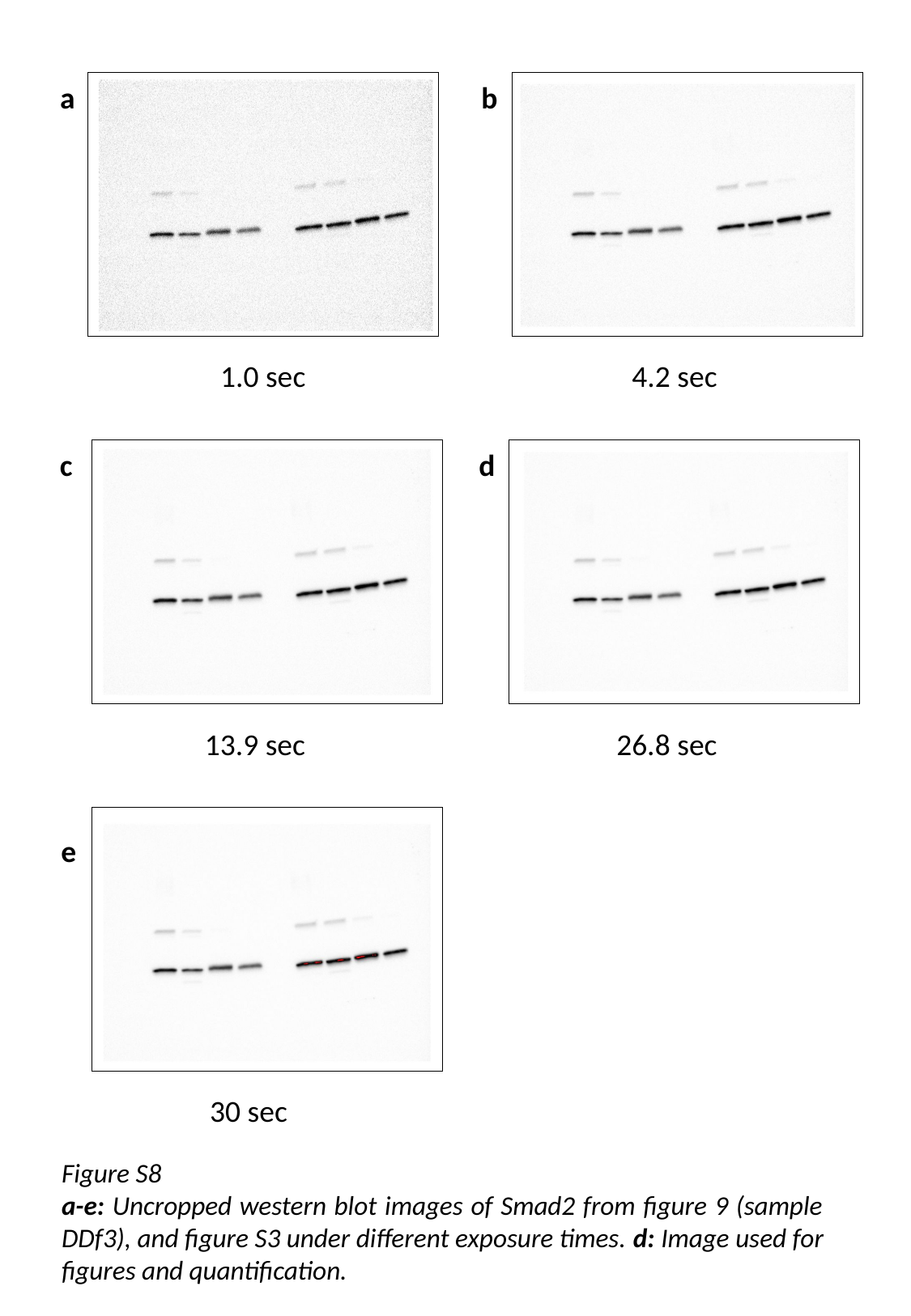

a
b
1.0 sec
4.2 sec
c
d
13.9 sec
26.8 sec
e
30 sec
Figure S8
a-e: Uncropped western blot images of Smad2 from figure 9 (sample DDf3), and figure S3 under different exposure times. d: Image used for figures and quantification.

## Slide 11
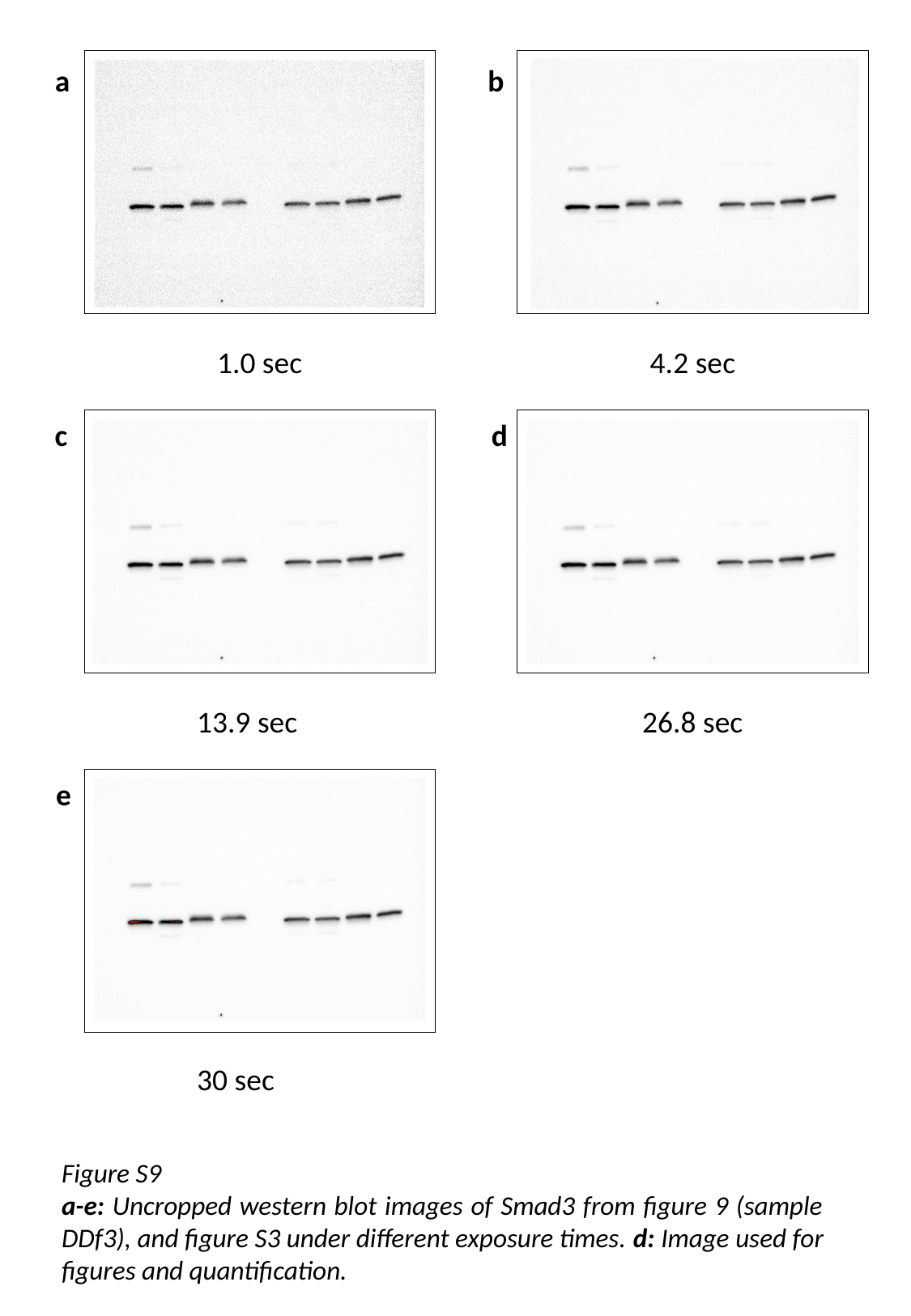

a
b
1.0 sec
4.2 sec
c
d
13.9 sec
26.8 sec
e
30 sec
Figure S9
a-e: Uncropped western blot images of Smad3 from figure 9 (sample DDf3), and figure S3 under different exposure times. d: Image used for figures and quantification.

## Slide 12
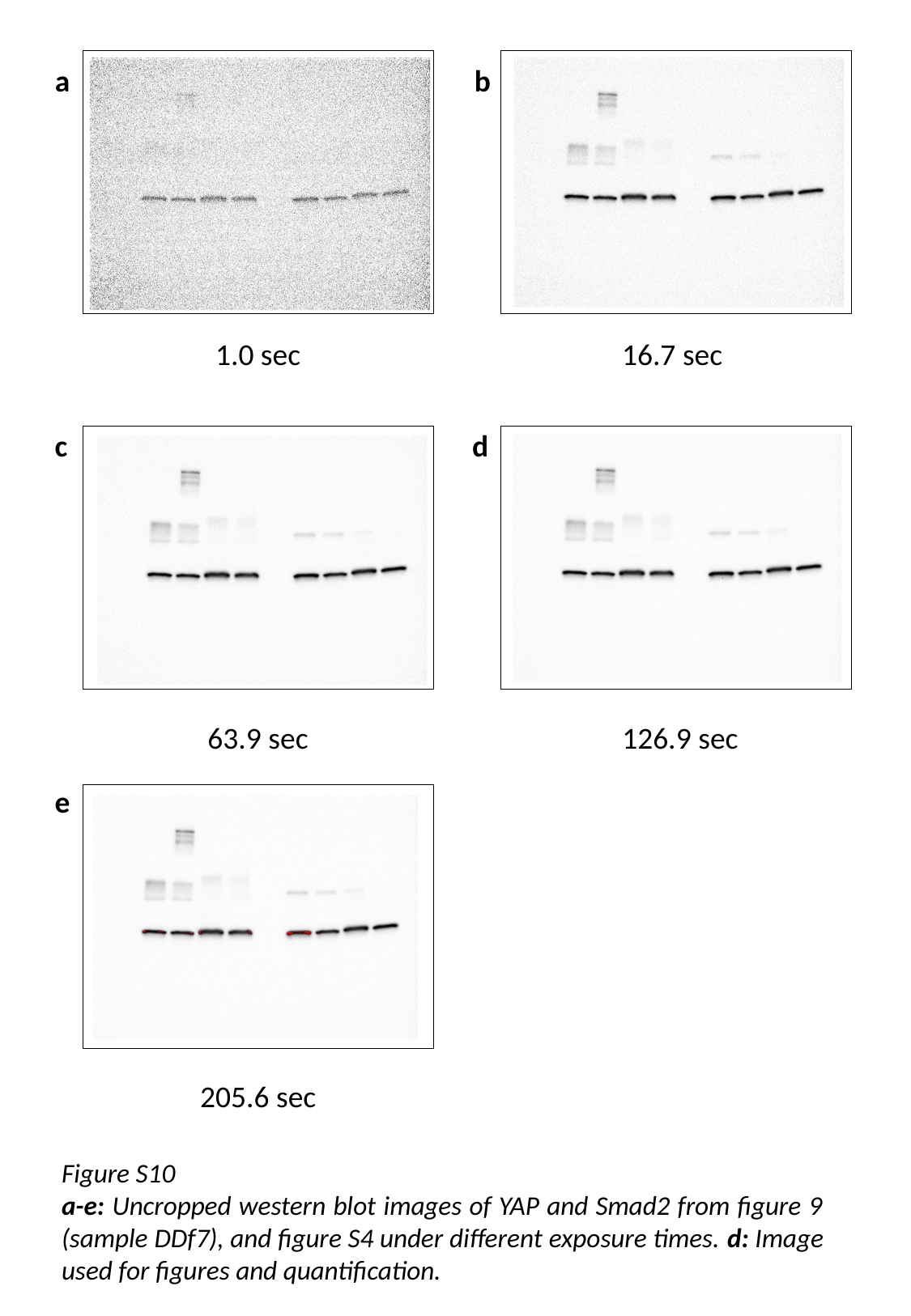

a
b
1.0 sec
16.7 sec
c
d
63.9 sec
126.9 sec
e
205.6 sec
Figure S10
a-e: Uncropped western blot images of YAP and Smad2 from figure 9 (sample DDf7), and figure S4 under different exposure times. d: Image used for figures and quantification.

## Slide 13
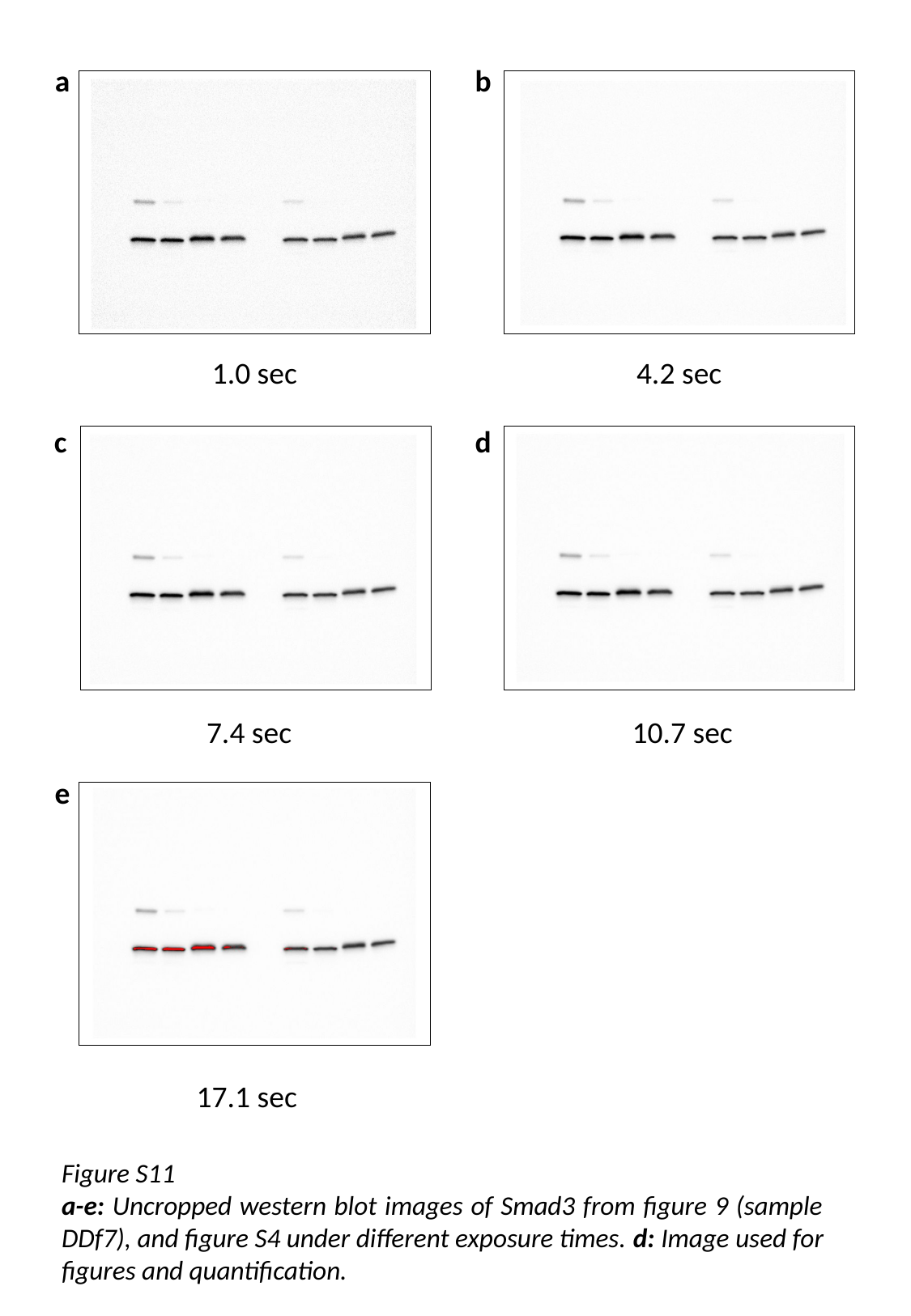

a
b
1.0 sec
4.2 sec
c
d
7.4 sec
10.7 sec
e
17.1 sec
Figure S11
a-e: Uncropped western blot images of Smad3 from figure 9 (sample DDf7), and figure S4 under different exposure times. d: Image used for figures and quantification.

## Slide 14
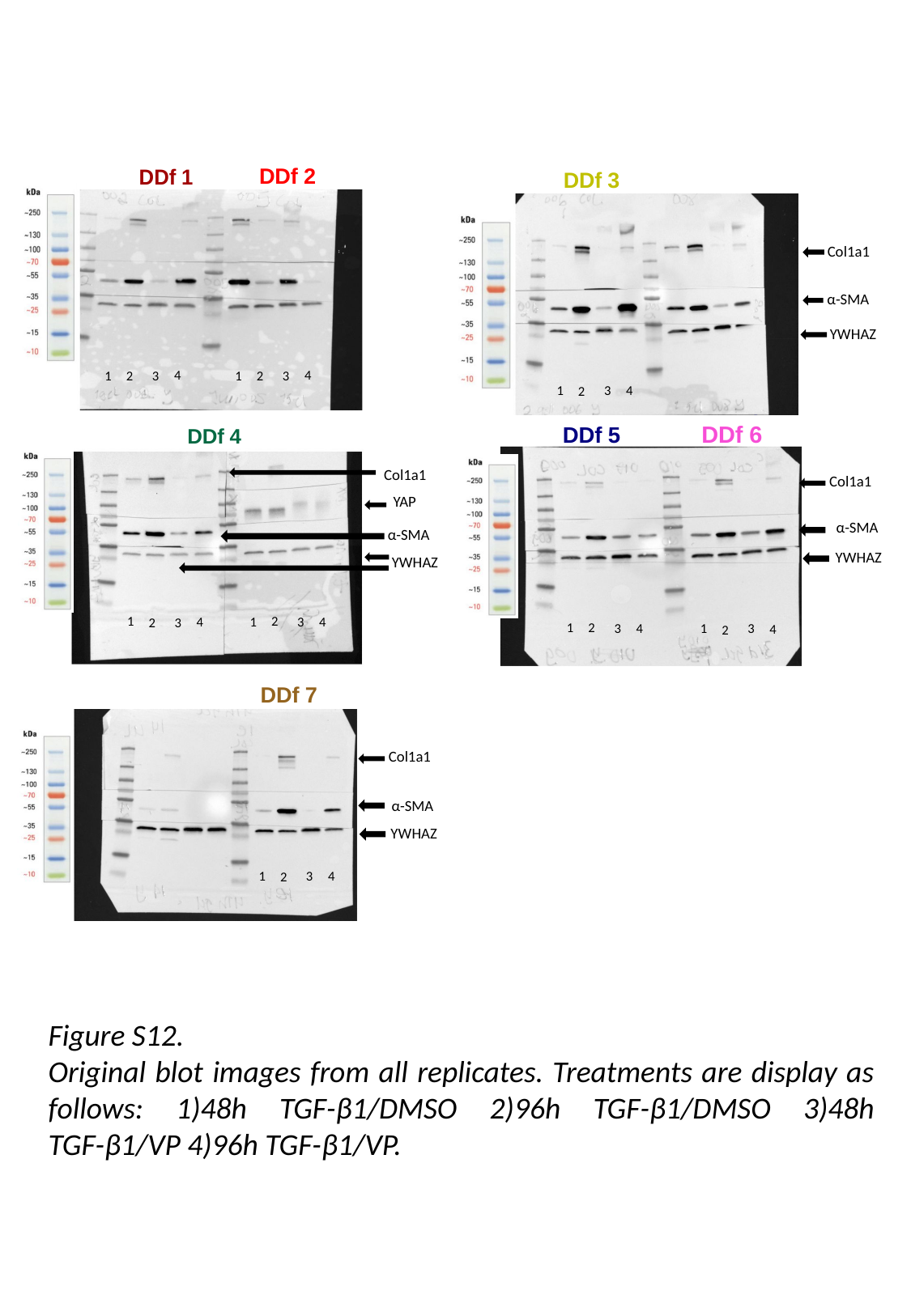

Col1a1
α-SMA
YWHAZ
4
4
2
2
1
1
3
3
1
3
4
2
Col1a1
Col1a1
YAP
α-SMA
α-SMA
YWHAZ
YWHAZ
1
2
1
4
3
4
2
3
2
1
3
4
1
3
4
2
Col1a1
α-SMA
YWHAZ
1
3
4
2
Figure S12.
Original blot images from all replicates. Treatments are display as follows: 1)48h TGF-β1/DMSO 2)96h TGF-β1/DMSO 3)48h TGF-β1/VP 4)96h TGF-β1/VP.

## Slide 15
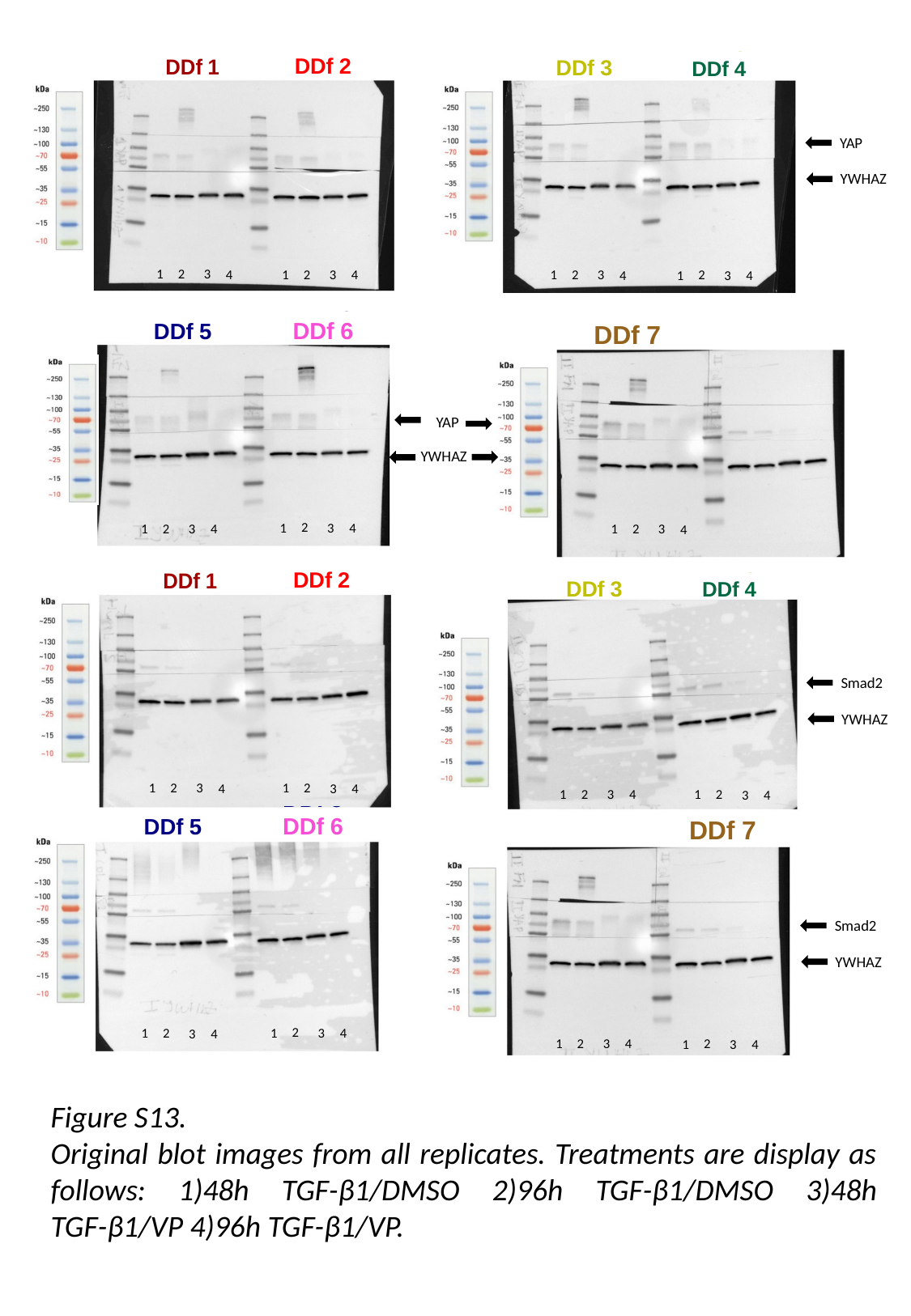

YAP
YWHAZ
2
1
3
2
4
1
2
3
1
2
4
3
1
4
3
4
YAP
YWHAZ
2
1
3
4
2
2
1
1
3
3
4
4
Smad2
YWHAZ
2
1
2
3
1
4
3
4
2
1
2
3
1
4
3
4
Smad2
YWHAZ
2
1
3
4
2
1
3
4
2
1
3
2
4
1
3
4
Figure S13.
Original blot images from all replicates. Treatments are display as follows: 1)48h TGF-β1/DMSO 2)96h TGF-β1/DMSO 3)48h TGF-β1/VP 4)96h TGF-β1/VP.

## Slide 16
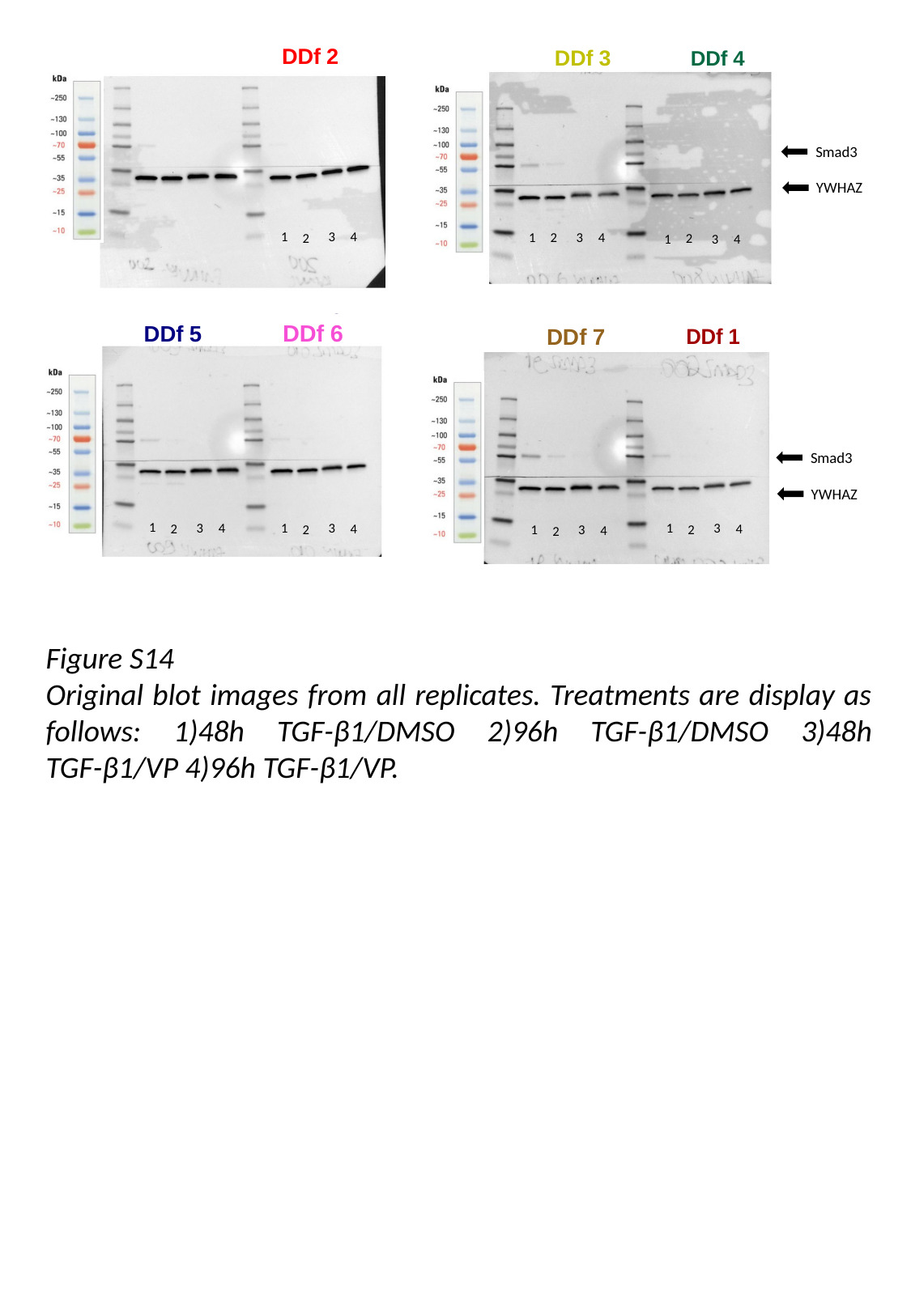

Smad3
YWHAZ
1
3
4
2
1
3
4
2
2
1
3
4
Smad3
YWHAZ
1
3
4
1
1
3
3
4
4
2
2
2
1
3
4
2
Figure S14
Original blot images from all replicates. Treatments are display as follows: 1)48h TGF-β1/DMSO 2)96h TGF-β1/DMSO 3)48h TGF-β1/VP 4)96h TGF-β1/VP.
